# A Flexible Implementation of Strong Segregation Theory for Two-Dimensional ABC Star Terpolymer Morphologies

**DOI:** 10.1021/acs.macromol.5c03113

**Published:** 2026-06-15

**Authors:** Merin Joseph, Daniel J. Read, Alastair M. Rucklidge

**Affiliations:** † Department of Chemistry, Technical University of Denmark, Kgs. Lyngby 2800, Denmark; ‡ Niels Bohr Institute, University of Copenhagen, Copenhagen 2100, Denmark; § School of Mathematics, 4468 University of Leeds, Leeds LS2 9JT, U.K.

## Abstract

We present a novel computational implementation of strong segregation theory, developed specifically for calculations of phase separated ABC star terpolymers. The method allows calculation of free energies of common two-dimensional morphologies for these polymers and the efficient construction of phase diagrams. The branch points of the ABC star terpolymers are localized in core regions, modeled as cylinders in three dimensions, and our framework is applicable to morphologies with single and multiple core types. Our central idea is that all the structures we wish to model can be assembled from a flexible base motif, which we call Strongly Segregated Polygons. This method is useful for exploring a wide range of complex morphologies, using a range of compositions and interaction strengths. We focus on 2D morphologies of ABC star terpolymers, but our method could be extended into three dimensions and to other molecular architectures, and in principle to large, irregular quasiperiodic two-dimensional structures.

## Graphical abstract

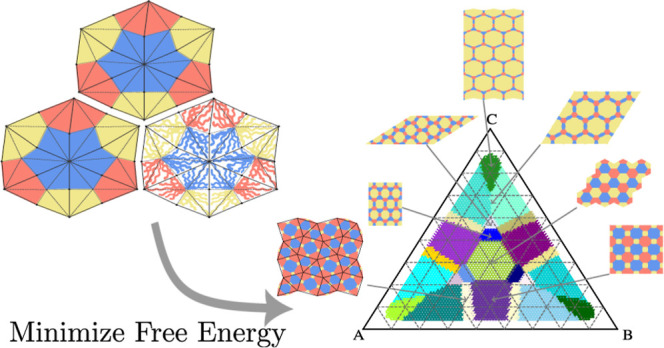

## Introduction

In this paper, we present a novel computational implementation of strong segregation theory, developed specifically for calculations of phase separated ABC star terpolymers. The method allows calculation of free energies of common morphologies for these polymers and the construction of phase diagrams, but (because of its relative simplicity and computational efficiency) is in principle extendable to large, irregular quasiperiodic two-dimensional (2D) structures. Here, we will present the details and development of the method and its application to simple 2D morphologies.

Strong segregation theory, first developed by Semenov,^1^ is one of several methods that have been applied successfully in the study of the rich variety of block copolymer morphologies. The theory is applicable when the repulsive interaction strength between monomers is large enough that blocks are stretched away from thin interfaces between regions of different monomer type. This gives rise to a balance between interfacial energy and stretching energy, which can be computed for various morphologies, allowing a phase diagram to be constructed. This analytical method was further extended to include bicontinuous structures^2–5^ for diblocks, polymer brushes^6^ and star-copolymers/miktoarms.^7^

When the interaction strength is lower, the stability of phase separated morphologies is classically studied using other methods: linear theory,^8^ weak segregation theory^9^ and self-consistent field theory.^10^ In these methods, monomer density fluctuations are taken into account in the free energy calculation. When the interaction strength is very low, the block copolymer melt is almost homogeneous, and weak segregation theory, first developed by Leibler,^9^ is often applied. In this limit, the phase separated structures are often described in terms of sinusoidal modulations in composition, retaining the lowest order interactions between different wavenumbers. For increased interaction strengths, self-consistent field theory (SCFT), first developed by Helfand et al.^11^ and later extended to diblocks by Matsen et al.,^12^ is used. Here, the monomer composition is treated as a spatially varying field and the configuration distribution of chains interacting within that field is determined. Yet, the monomer composition must be self-consistent with the chain configurations, so the solution is iterated until the composition field and chain configurations agree. The SCFT framework is the main workhorse for the study of microphase separation owing to its accuracy and validity in the intermediate and strong segregation limits:^13–16^ it essentially overlaps with and bridges between the strong and weak segregation limits. The open-access PSCF software^13^ is widely used to implement SCFT calculations for a variety of block copolymer systems.

All three nonlinear methods agree in the ordering of classical diblock morphologies (lamellae, rods, gyroid and spheres) in the simple diblock case.^2,9,10^ It is nevertheless computationally intensive to perform SCFT calculations, especially as the molecular complexity and size of the simulation box increases.^17,18^ Under these circumstances, it remains useful to return to simpler theories, such as strong segregation theory, to screen a wide variety of compositions and interaction strengths before undertaking SCFT calculations. By developing a simple computational implementation of strong segregation theory, we are here aiming toward a useful tool that will allow a wide variety of morphologies to be investigated with relative computational ease.

ABC star terpolymers are synthesized by grafting a third block (of type C) to a diblock at the junction where the A and B blocks meet.^19–25^ This results in a branched block copolymer, illustrated in Figure 1a. If the degree of polymerization of all three blocks is sufficiently large and the chemistries sufficiently incompatible, then melts of such molecules tend to microphase separate into regions rich in each of the A, B or C blocks.^20,26,27^ This results in a wide variety of phases that have been investigated experimentally, computationally and theoretically. We present a summary of experimental results in Table 1 and computational/theoretical results in Table 2.

**Figure 1:**
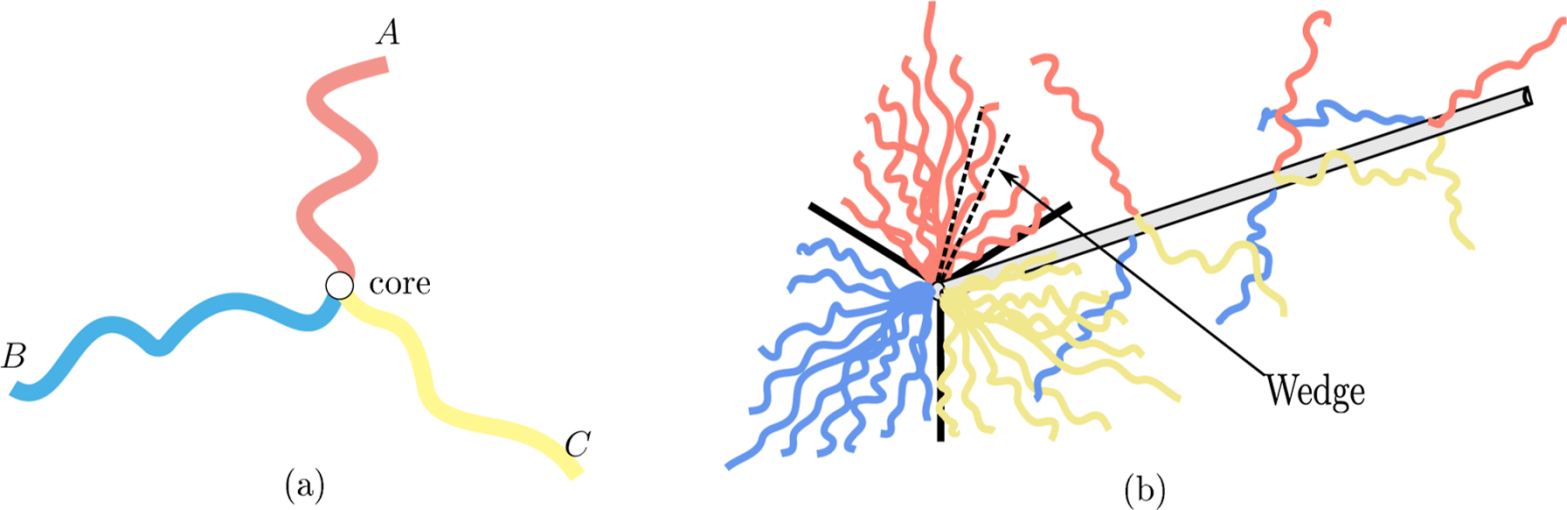
A graphical representation of (a) an ABC star terpolymer and (b) phase separation in ABC star terpolymer melts. The three branches (red, blue and yellow) join at their core, and in three dimensions, the different polymer types collect into three domains, with the cores aligned in a straight line, forming columnar structures in one dimension and ordered geometrical patterns in the other two. The cores form a structure that can be taken as a narrow cylinder (gray) to which the polymer branches are grafted. The radius of this cylinder is comparable to the length of a monomer unit. The interfaces between the three domains are indicated by thick black lines. The stretching free energy is calculated by slicing each domain into small wedges, indicated by dashed lines.

**Table 1:** A Summary of Morphologies Reported Experimentally in ABC Star Terpolymer Melts

article	polymer types	morphologies identified
Hadjichristidis et al.^29^	PS, PI, PB	Cylinders of PS and miscible matrix of PI and PB
Sioula et al.^27,30^	PS, PI, PMMA	Coaxial cylinders in 2D hexagonal lattice
Okamoto et al.^26^	PS, PDMS, PTBMA	[6,6,6]
Hückstädt et al.^24^	PS, PB, PVP	[8.8.4], [12.6.4], Lamellar + Coaxial cylinder
Yamauchi et al.^31^	PS, PI, PDMS	[8.8.4]
Takano et al.^32,33^	Blends of PI, PS, PVP	[6.6.6], [8.8.4], [12.6.4], [10.8.4; 10.6.4]
Hayashida et al.^34,35^	PI, PS, PVP blends	Dodecagonal quasicrystals and [10.8.4; 10.6.4]
Aissou et al.^36^ and Nunns et al.^37^	PI, PS, PISF	[8.8.4], [6.6.6]
Choi et al.^38^	PI, PS, PISF	L + C
Chernyy et al.^39^	PDMS, PI, PMMA	[6.6.6], [8.8.4], L + C and columnar discs
Ariaee et al.^40^	PI, PS, PMMA	L + C, Coaxial cylinders in 2D hexagonal lattice, Coaxial cylinders in 2D square lattice

**Table 2:** A Summary of Morphologies in ABC Star Terpolymers Reported Using Different Computational Methods

article	computational framework	morphologies identified
Dotera et al.^41^	Monte Carlo (Diagonal Bond Method)	[6.6.6]
Bohbot et al.^42^	SCFT	[6.6.6], [8.8.4]
Gemma et al.^43^	Monte Carlo (Diagonal Bond Method)	Lamella + sphere (L + S), [8.8.4], [6.6.6], [8.6.4; 8.4.6; 8.6.6], [10.6.4; 10.4.6; 10.6.6], [12.6.4], perforated layer (PL), L + C, columnar piled disk (CPD), and lamella-in-sphere (L-in-S)
He et al.^44,45^	Dynamic Density Functional Theory	[6.6.6], [8.4.4], [12.6.4], coaxial cylinders, lamellae
Ueda et al.^46^	Monte Carlo (Diagonal Bond Method)	[6.6.6], [8.4.4], [12.6.4], coaxial cylinders, L + S, L + C, [10.8.4; 10.6.4]
Birshtein et al.^47^	SST	Lamellae
Dotera et al.^48^	Monte Carlo (Diagonal Bond Method)	12-fold quasicrystals
Huang et al.^49^	Dissipative Particle Dynamics (DPD)	[6.6.6], [8.4.4], [12.6.4], coaxial cylinders, L + S, L + C, [10.8.4; 10.6.4], disordered networks
Li et al.^18^	SCFT	L + S, [8.8.4], [6.6.6], [8.6.4; 8.4.6; 8.6.6], [10.6.4; 10.4.6; 10.6.6], [12.6.4], [10.8.4; 10.6.4], PL, L + C, CPD
Zhang et al.^50^	SCFT	[8.8.4], [6.6.6], [8.6.4; 8.4.6; 8.6.6], [10.6.4; 10.4.6; 10.6.6], [12.6.4], [8.6.4; 8.8.4; 12.6.4; 12.8.4], L + C, lamellae
Xu et al.^51^	SCFT	[8.8.4], [6.6.6], [8.6.4; 8.4.6; 8.6.6], [10.6.4; 10.4.6; 10.6.6], [12.6.4], [8.6.4; 8.8.4; 12.6.4; 12.8.4], [10.8.4; 10.6.4], L + C, lamellae and disordered networks
Kirkensgaard et al.^52^	Dissipative Particle Dynamics (DPD)	[8.8.4], [6.6.6], [8.6.4; 8.4.6; 8.6.6], [10.6.4; 10.4.6; 10.6.6], [12.6.4], [12.6.6; 12.6.4; 12.4.4], [10.8.4; 10.6.4], L + C, lamellae
Jiang et al.^53^	SCFT	[8.8.4], [6.6.6], [8.6.4; 8.4.6; 8.6.6], [10.6.4; 10.4.6; 10.6.6], [12.6.4], [8.6.4; 8.8.4; 12.6.4; 12.8.4], [10.8.4; 10.6.4], L + C, lamellae, disordered networks
Hawthorne et al.^16^	SCFT	[8.8.4], [6.6.6], [8.6.4; 8.4.6; 8.6.6], [10.6.4; 10.4.6; 10.6.6], [12.6.4], [8.6.4; 8.8.4; 12.6.4; 12.8.4], [10.8.4; 10.6.4], L + C, lamellae, cylinders

When *A*-, *B*- or *C*-rich domains are strongly segregated, molecules must be configured in such a way that the A, B and C blocks can extend into their respective domains. Hence, the junction point of the stars must be located at places where all three domain types meet. The meeting of three domains in 3D space will trace out a space curve, so we anticipate that junction points of stars are located at these space curves, as illustrated in Figure 1b. We expect a large number of star junction points to be located along each of these curves and will henceforth refer to these curves as the “core” regions of the structures.

In many (but not all) cases, structures formed are quasi-2D in nature, where a phase-separated pattern in 2D is extended, unchanged, into the third dimension. In such cases, the “cores” are points in 2D where three domain types meet, and form straight lines in the third dimension (see Figure 1b). These quasi-2D structures will be the main focus of this paper. They can be depicted as 2D tilings of A-, B- and C-rich regions, with the areas of the different domains related to the degree of polymerization of each monomer type. Since these tilings must contain the cores at the intersection of all three domains, each domain type (e.g., *A*) must be surrounded by alternating domains of the other two types (e.g., *B* and *C*) with the cores located at the points (in 2D) where all three domain types meet.

Even with this restriction, a rich variety of phases is possible, and several of the commonly investigated periodic structures are illustrated in Figure 2. The commonly used notation for most of these uses Schläfli symbols,^28^ which are triplets of three numbers [*x*,*y*,*z*] indicating the structure. These numbers refer to the number of neighboring domains surrounding each domain type. The simplest structures are depicted by only one triplet of numbers, e.g., [8.8.4] is a morphology with square symmetry in which two of the domain types (e.g., *A* and *B*) are surrounded each by eight neighboring domains, while the third type (e.g., *C*) is surrounded only by four (see Figure 2). Morphologies [6.6.6] and [12.6.4] similarly require only a single triplet of numbers. These structures are simple in the sense that all cores in the structure are identical, being surrounded by equivalent arrangements of *A*, *B* and *C* domains. The “L + C” (lamellae + cylinder) phase depicted in Figure 2 is also simple in the same sense (having only one core type) but is not usually denoted by the triplet notation since the lamellar domains are surrounded by an infinite number of neighbors.

**Figure 2:**
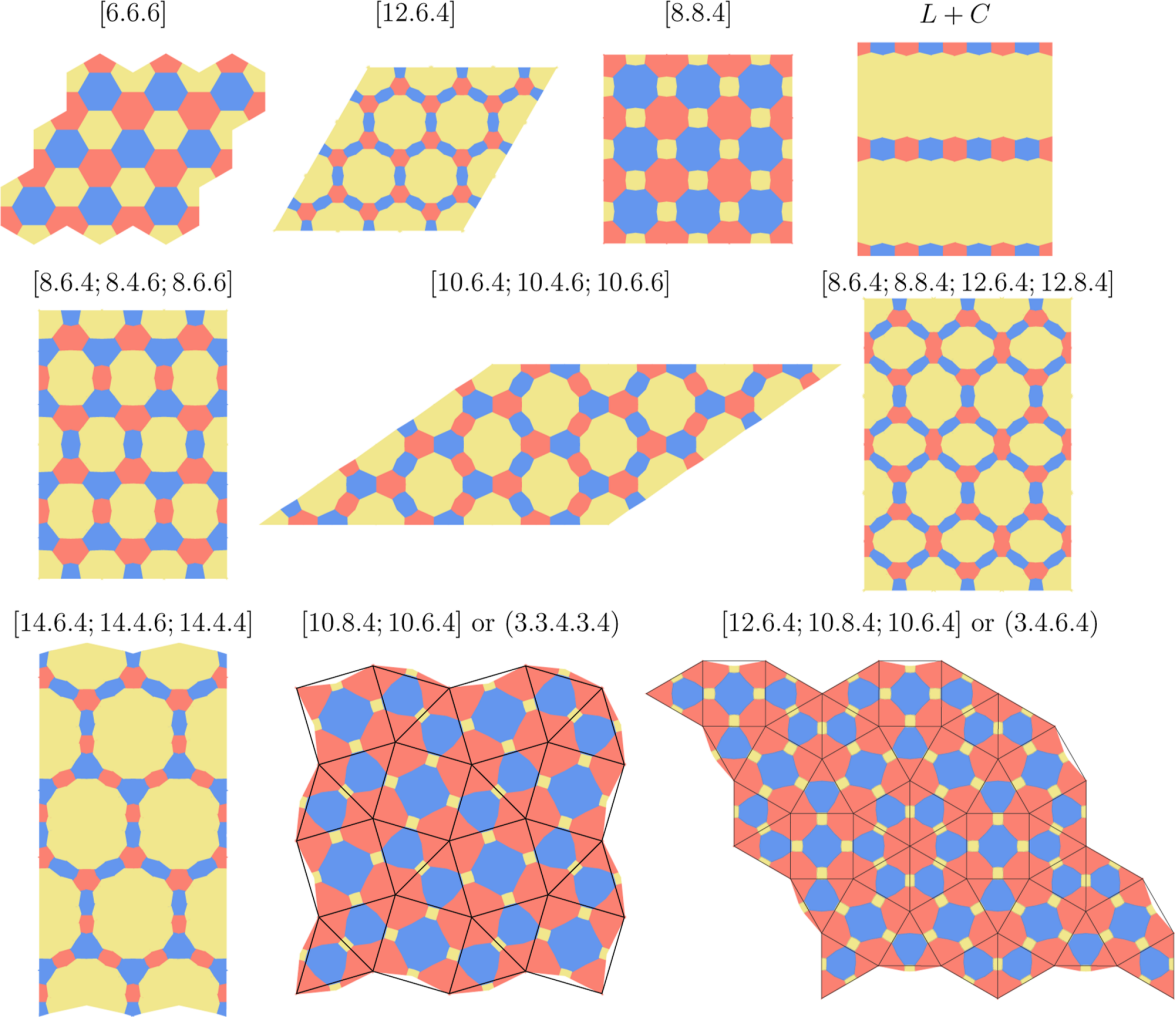
All candidate morphologies considered in this work are illustrated here. The color scheme is *A* (red), *B* (blue) and *C* (yellow), and the areas of each domain are related to the lengths of the *A*, *B* and *C* branches. The cores are at the junctions of the red, blue and yellow regions. The morphologies are all periodic in space, and several repeats of the periodic morphologies are shown (four repeats in the last two examples). The last two morphologies are decorated (in black) with square and triangular tiles, which assist in constructing the morphologies.

More complicated structures are denoted by multiple triplets because there are multiple types of domain for each given monomer. As a result, these structures also contain multiple types of core that are not equivalent. One way of labeling these is to consider the three domains adjacent to each core, and to note the number of neighbors connected to each of those three neighboring domains. For example, we denote a three-core structure by [8.6.4; 8.4.6; 8.6.6]; see Figures 2 and 5 for more detail. This structure contains some cores adjacent to an 8-neighbor *C* domain, 6-neighbor *B* domain and 4-neighbor A domain; some cores adjacent to an 8-neighbor *C* domain, 4-neighbor *B* domain and 6-neighbor *A* domain; and some cores adjacent to an 8-neighbor *C* domain, 6-neighbor *B* domain and 6-neighbor *A* domain. Although in this case it is the *C* domains that have 8 neighbors, the standard Schläfli notation is to list the highest number first, regardless of the polymer type. Even this notation is not exhaustive: there are actually two different types of [8.6.6] core, not equivalent by symmetry, within this structure.

Another important structure is the so-called “Σ-phase”, also denoted by the Archimedian tiling notation (3.3.4.3.4),^28^ which we label as [10.8.4; 10.6.4], while noting that there are several types of each of the [10.8.4] and [10.6.4] cores not equivalent by symmetry. As we will see below, the fact that there are multiple core types within a single structure is an important consideration when minimizing the energy: different core types will typically contain a different number of star centers per unit length.

ABC star terpolymers have been synthesized from different monomers, and phase separated morphologies are observed in most examples. We give a sample of these in Table 1. The majority of morphologies found in this wide range of experiments are single core. Multicore structures typically require blends of ABC star terpolymers with other components. Table 2 reports a sample of computational and theoretical studies of morphologies of ABC star terpolymers. These studies report a variety of quasi-2D morphologies and some 3D morphologies, finding a wider range than identified in experiments.

In this paper, we focus specifically on the 2D morphologies of ABC star terpolymer melts, and particularly on developing a computational framework for implementing a strong segregation theory for these morphologies. Previously, Gemma et al.^43^ constructed a strong segregation theory (SST) for some specific structures, those with a single core type. In our work, we aim to generalize this, creating a framework that is applicable to morphologies with multiple core types, more disordered patterns, or quasicrystalline approximants. Key to all these structures is the existence of cores, as described above, with the star arms strongly stretched away from the cores into their respective *A*-, *B*-, or *C*-rich domains.

Our central idea is that all the structures we wish to model can be assembled from a flexible base motif structure, illustrated in Figure 3a below. We call these motif structures “strongly segregated polygons” (SSPs). Each SSP contains a single core, surrounded by *A*, *B* and *C* domains, and we represent its shape by a polygon. The minimal number of edges is six, giving a balance between flexibility and complexity. Each six-sided SSP requires six outer nodes to specify its shape, so that it tessellates with the other polygons to form the overall morphology. In our method, we tessellate a periodic domain with SSPs, compute the free energy of the configuration, and minimize this free energy by adjusting the positions of the boundary points of the SSPs. In structures with only one core type, all these SSPs are generally identical (up to symmetry) in a minimal energy structure. However, in more complicated structures with multiple core types, there will naturally be variation between the SSPs, both in shape and area.

**Figure 3:**
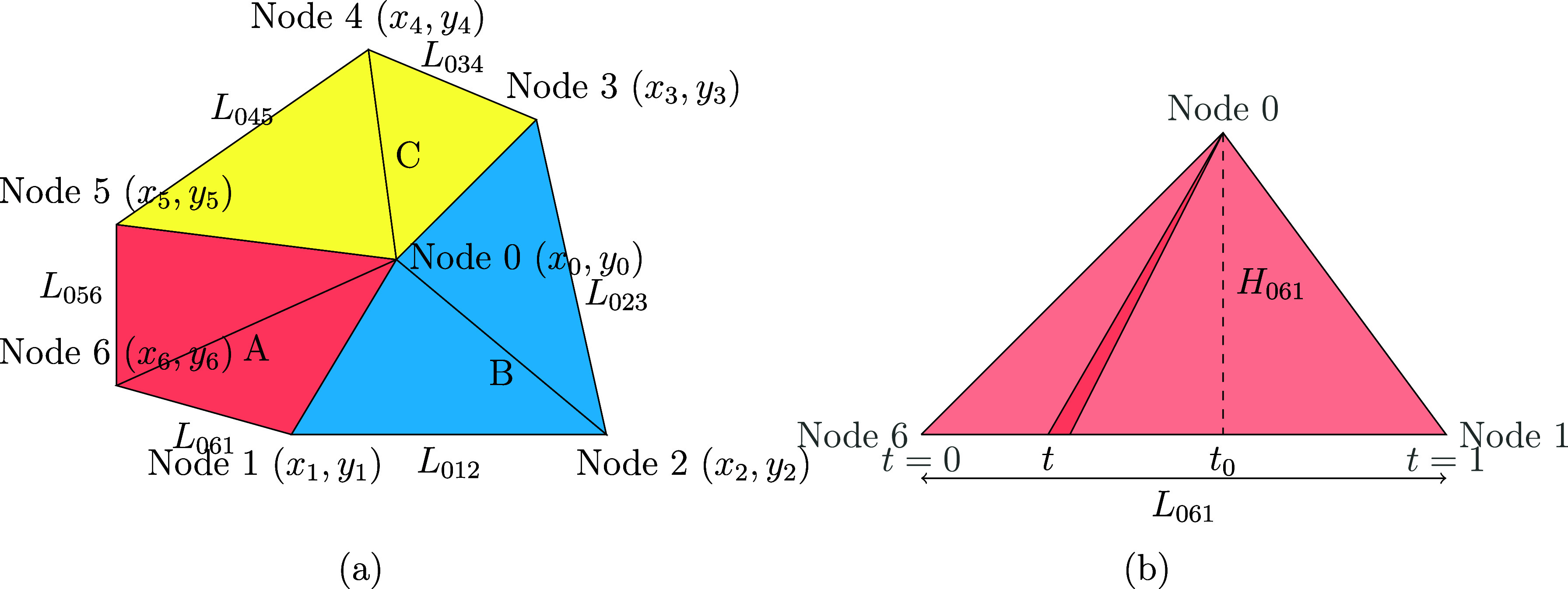
Geometrical structure of a Strongly Segregated Polygon (SSP). In (a), the six-sided SSP has one node (Node 0) in the center and six nodes around the edges. The three colors indicate the regions of three types of polymer. In (b), we show triangle 061 of the SSP, with a coordinate *t* that runs from 0 to 1 along the bottom of the triangle. The dimensionless height of the triangle is *H*_061_ and the length of its base is *L*_061_. A wedge (at position *t*) within this triangle is illustrated.

We anticipate that this method will be useful for exploring a wide range of complex morphologies, using a range of compositions and interaction strengths. While SST is only valid in the limit of high interaction strengths, the distribution of domains of monomer types can be used as initial conditions for more accurate calculations using (for example) SCFT. We focus on 2D morphologies of ABC star terpolymers, but our SSP method could be extended into three dimensions and to other molecular architectures. As an example of this for AB block copolymers, Reddy, Dimitriyev and Grason^4,5^ constructed a strong segregation theory for bicontinuous phases such as double-gyroid, in which the interconnected structure is composed of “meso-atoms”. To compute the chain conformations and free energy within each meso-atom, they generalized the idea of “wedges” used previously by Olmsted and Milner.^2^ Each wedge comprises an extended volume terminating in two triangular faces, with an internal triangular facet separating the *A* and *B* rich domains, such that the A and B blocks stretch away from the internal facet. Hence, the wedge is the tessellating base motif structure for an AB copolymer, analogous to our SSPs.

The rest of this paper is devoted to establishing the free energy for a single SSP; demonstrating that candidate morphologies can be constructed from an assembly of SSPs; minimizing the free energy of each morphology by moving the nodes of each SSP; and creating phase diagrams for the ABC star terpolymer melt with several choices of parameter values.

## Methodology

In the strong segregation limit, the free energy of a branched block copolymer system depends only on the geometry of the domains of the different blocks and the repulsion between monomer units of different types. The calculations needed for strong segregation theory are relatively easy (compared to other mean field approximations) and can be carried out for a wide variety of morphologies.^54^

### Free Energy Calculation

In this work, we focus on ABC star terpolymers as illustrated in Figure 1a, with the A, B and C branches joining at a core. We assume that the cores of the polymer molecules align in straight lines, with domains of *A*, *B* and *C* surrounding each line, giving a two-dimensional pattern when viewed from the third direction. We consider a line of cores as a cylinder in three dimensions, of radius *R*_core_ expected to be comparable to the monomer size *b*, with the three polymer types grafted to the cylinder, as shown in Figure 1b. We separate the free energy calculation into three parts: the stretching energy for chains outside the core region; consideration of the core itself; and interfacial energy due to surface tension between the *A*, *B* and *C* domains.

In order to evaluate the stretching energy of the polymer branches, we divide the three regions into wedges, as shown in Figure 1b, with wedges containing polymers grafted onto the inner cylinder. The stretching energy for polymer brushes grafted onto a convex surface of radius of curvature *R*_core_ is reported by Ball et al.^55^ In a wedge of height *h* containing monomers of type *I* in blocks of length *N*_*I*_, the stretching free energy per chain in units of *k*_*B*_ *T* is

1 $$\frac{3}{4}\frac{h^{2}}{N_{I}b^{2}}\log\frac{h^{2}}{R_{core}^{2}}$$

where we have taken the leading term in the limit of small *R*_core_.^55,56^ For convenience, from this point onward, we express all lengths in units of $R=\sqrt{Nb^{2}}$ , where *N* = *N*_*A*_ + *N*_*B*_ + *N*_*C*_ is the total number of monomers in a star, and we define ϕ_*I*_ = *N*_*I*_/*N*. Then the stretching energy per chain in a wedge becomes

2 $$f_{chain}\left(H,\phi_{I}\right)=\frac{3}{4\phi_{I}}H^{2}\;\log\left(cH^{2}\right)=\frac{3}{4\phi_{I}}F_{chain}\left(H\right)$$

where *H* = *h*/*R*, *c* = *R* ^2^/*R*_core_^2^ and *F*_chain_(*H*) = *H* ^2^ log­(*cH* ^2^). The stretching free energy of the whole morphology can then be found by summing over all the wedges with different heights and different monomer types.

In order to evaluate this sum, we will describe the two-dimensional morphologies formed by ABC terpolymers in terms of polygons, with a core cylinder at the center of each polygon. One such Strongly Segregated Polygon (SSP) is shown in Figure 3a: it is made up three domains, one for each polymer type, with the domains divided in two, resulting in six triangles. We assume that all polymers within each domain are attached to the core at Node 0 and stretched toward the edge of the domain, and that the number of polymers in a wedge is proportional to the area of the wedge. The stretching free energy per chain in each triangle is then the integral over the wedges making up that triangle (Figure 3b). The interfacial energy comes from the boundaries between the domains of the three different polymer types within each SSP. There is no interfacial energy contribution between two SSPs because we assemble these so that they join at regions containing the same monomer type.

The stretching free energy, interfacial energy and core energy of one SSP depend on the location of all its vertices, the monomer compositions (ϕ_*A*_, ϕ_*B*_ and ϕ_*C*_) and the interaction strengths (expressed in terms of the Flory interaction parameters as *N*χ_*AB*_, *N*χ_*BC*_ and *N*χ_*AC*_). Given the positions of Nodes 1 to 6, the position of Node 0 at the center is determined uniquely from the monomer compositions, with the constraint that Node 0 should lie within the polygon (see eq S3 in the Supporting Information).

To determine the stretching energy per chain for a single triangle, we divide the triangle into wedges (see Figure 3b). We give the outline of the calculation here, with further details in the Supporting Information. We take the 061 triangle as an example, containing polymer A. This triangle has base length *L*_061_ and height *H*_061_, measured in units of $R=\sqrt{Nb^{2}}$ . The triangle has a coordinate *t* that runs from 0 to 1 along its base, and *t*_0_ gives the location of the perpendicular projection of node 0 onto the base. The height of a wedge (in units of *R*) from node 0 to the point parametrized by *t* is $H\left(t\right)=\sqrt{{\left(t-t_{0}\right)}^{2}L_{061}^{2}+H_{061}^{2}}$ . The stretching energy per chain for the whole triangle is then

3 $$f_{061}=\frac{A_{061}}{\phi_{A}A_{T}}\int_{0}^{1}f_{chain}\left(H\left(t\right),\phi_{A}\right)dt=\frac{3A_{061}}{4\phi_{A}^{2}A_{T}}\int_{0}^{1}F_{chain}\left(H\left(t\right)\right)dt$$

where *A*_061_ is the area of the triangle and *A*_*T*_ is the area of whole SSP, in units of *R* ^2^. The function *F*_chain_(*H*) = *H* ^2^ log­(*cH* ^2^) and *c* = *R* ^2^/*R*_core_^2^ from above.

The second integral in eq 3 can be written explicitly as

4 $$I_{061}\left(X,Y,t_{0}\right)=\int_{0}^{1}\left({\left(t-t_{0}\right)}^{2}X+Y\right)\log\left(c\left({\left(t-t_{0}\right)}^{2}X+Y\right)\right)dt=\log\left(c\left(X{\left(1-t_{0}\right)}^{2}+Y\right)\right)\left(\frac{X{\left(1-t_{0}\right)}^{3}}{3}+Y\left(1-t_{0}\right)\right)+\log\left(c\left(Xt_{0}^{2}+Y\right)\right)\left(\frac{Xt_{0}^{3}}{3}+Yt_{0}\right)-\frac{2X}{9}\left({\left(1-t_{0}\right)}^{3}+t_{0}^{3}\right)-\frac{4Y}{3}+\frac{4Y}{3}\sqrt{\frac{Y}{X}}\left(\arctan\sqrt{\frac{X}{Y}}\left(1-t_{0}\right)+\arctan\sqrt{\frac{X}{Y}}t_{0}\right)$$

where *X* = *L*_061_^2^, *Y* = *H*_061_^2^ and *c* = *R* ^2^/*R*_core_^2^. The stretching free energies for the remaining five triangles are calculated in a similar manner: triangle 0*ij*, between nodes 0, *i* and *j*, has base length *L*_0*ij*_ and height *H*_0*ij*_, so the stretching free energy is *f*_0*ij*_. The stretching free energy per chain of the entire SSP, in units of *k*_*B*_ *T*, is then

5 $$f_{str}=\frac{3}{4}\frac{1}{A_{T}}\left(\frac{A_{061}I_{061}+A_{056}I_{056}}{\phi_{A}^{2}}+\frac{A_{023}I_{023}+A_{012}I_{012}}{\phi_{B}^{2}}+\frac{A_{045}I_{045}+A_{034}I_{034}}{\phi_{C}^{2}}\right)$$

where, as in Figure 3a, triangles 061 and 056 contain polymer *A*, triangles 023 and 012 contain polymer *B*, and triangles 045 and 034 contain polymer *C*.

So far we have not considered the free energy of the core region itself, nor specified how large its radius *R*_core_ might be. Since we expect the core region to be of order of the monomer dimension, the exact details must depend on the specific chemistry, both of the monomer type in each of the three arms, and of the chemical unit used to form the branch point itself. Hence, it is not possible to develop a “universal” theory. However, we can make reasonable assumptions to arrive at a plausible first-order description of the core. We assume that the chains in the core region are sufficiently closely packed together so that the arms are forced to exit the core region as quickly as possible, i.e., they are strongly stretched away from the core at the monomer scale. Thus, if the core region contains *N*_core_ ≪ *N* monomers per chain, we expect *R*_core_ ≈ *N*_core_ *b*/3 for a three arm star. By equating the volume π*R*_core_^2^ *d* of a core section of length *d* with the volume occupied by the *N*_core_ monomers per chain (see the Supporting Information) we arrive at the estimates

$$\begin{array}{ll} N_{core} & \approx \frac{9A_{T}}{\pi }\\ R_{core} & \approx \frac{3A_{T}b}{\pi } \end{array}$$

where, as above, *A*_*T*_ is the area of whole SSP, in units of *R* ^2^. As *A*_*T*_ increases, the number of chains per unit length inside the core region also increases, so that the radius of the core region (in which chains are stretched to monomer level by chain packing) must increase. Hence, we find

6 $$c=\frac{R^{2}}{R_{core}^{2}}\approx \frac{\pi^{2}N}{9A_{T}^{2}}$$

i.e., the value of *c* used in eq 4 above is not constant but varies with the SSP area, and depends on the degree of polymerization *N* as well as the SSP geometry via the normalized area *A*_*T*_.

This dependence on *N* should be briefly commented on. In most theories of polymer phase separation, the dependence on *N* arises in combination with the interaction parameters χ, so that the product *N*χ is the relevant parameter. In our theory, *N*χ remains the leading order parameter, emerging as described below in the interfacial energy. The further dependence on *N* (independent of the interaction parameters χ) arises because of the explicit dependence on *R*_core_ in eq 1, giving a logarithmic dependence on *N* in the stretching energy via the parameter *c* in eq 4. As a result, varying *N* has a weak effect on the balance between stretching and interfacial energies, giving rise to small changes in the phase diagram, as we will describe below.

We expect the free energy for the core region to result from two main contributions: a stretching energy of order *k*_*B*_ *T* per monomer from stretching the chains at the monomer scale, and a mixing energy arising from forcing the A, B and C monomers into close proximity in the core region. Thus, we estimate the core energy per chain, in units of *k*_*B*_ *T*, to be proportional to *N*_core_ = 9*A*_*T*_/π with form

7 $$f_{core}=\frac{9A_{T}}{\pi }\left(s_{core}+\frac{\chi_{AB}+\chi_{BC}+\chi_{AC}}{9}\right)$$

where *s*_core_ is an order one parameter related to the change in free energy, in units of *k*_*B*_ *T*, for fully orienting a single monomer in the direction away from the core. To obtain the second “mixing” term inside the brackets, we assume the core to be comprised of equal fractions of all three monomer species. Since *A*_*T*_ ∼ *H* ^2^, where *H* is the (nondimensional) degree of stretching of the chains in the SSP, this core energy acts in a largely similar way to the stretching energy. Since *s*_core_ is of order one, and since experimentally reported Flory interaction parameters (χ) in the ABC terpolymer systems listed in Table 1 have values less than 0.1, we take

8 $$f_{core}=\frac{9}{\pi }A_{T}$$

in all calculations reported in this paper.

Although the above description of the core region is necessarily approximate, we believe none of our results are sensitive to the approximations used. Indeed, setting *c* to be a reasonable constant, and setting *f*_core_ = 0, results in the same geometry of phase diagrams and same form of free-energy minimized morphologies as we present below. This is because any quantitive changes are similar across all morphologies.

The interfacial energies at the three interfaces, AB, BC and AC, contribute to the interaction free energy. The Flory interaction parameter χ is proportional to the square of the surface tension γ at the interfacial plane.^57^ The interfacial energy per chain *f*_int_ in units of *k*_*B*_ *T* is then

9 $$f_{\mathrm{int}}=\frac{\gamma_{AB}L_{01}+\gamma_{BC}L_{03}+\gamma_{AC}L_{05}}{A_{T}}$$

where the interfaces have lengths (in units of *R*) *L*_01_ between *A* and *B*, *L*_03_ between *B* and *C*, and *L*_05_ between *A* and *C* (see Figure 3), and the three scaled surfaces tensions are γ_*AB*_, γ_*BC*_ and γ_*AC*_. These are related to the Flory interaction parameters by

10 $$\gamma_{IJ}=\sqrt{\frac{N\chi_{IJ}}{6}}$$

See the Supporting Information for details.

The total free energy per chain for an SSP *f*_*c*_ in units of *k*_*B*_ *T* is

11 $$f_{c}=f_{\mathrm{int}}+f_{core}+f_{str}$$

This free energy for a single SSP is thus a function of monomer composition, interaction strengths and node coordinates. A given morphology is initially set up as a combination of SSPs, and then the node coordinates can be adjusted to find a configuration that minimizes the total free energy per chain for the chosen monomer compositions and interaction strengths.

The discussion so far has been for six-sided SSPs, which we use for the majority of our calculations. However, lamellar phases (in particular, the L + C phase) cannot be represented with six-sided SSPs, so we extend the SSP method to allow for eight-sided SSPs, in which one of the domains is represented by four triangles, with two other domains represented with two triangles as before. The calculation of the stretching free energy within each triangle remains as before.

### Implementing the SSP Method

A required morphology is constructed using these SSPs by joining several of them together. In joining two SSPs, a region containing polymer type *A* in one SSP will join to a region containing type *A* in the other; similarly *B* and *C*. In addition, nodes are shared between two or more adjacent SSPs, and a change in location of a node will affect all SSPs that it belongs to. We are always concerned with morphologies that are periodic, so the matching of polymers and the identification of nodes carries across the sides of the periodic domain. For example, an extreme hexagonal morphology, [6.6.6], is created by joining six SSPs together, as shown in Figure 4a. The periodicity is indicated in the figure by matching colored lines along the outer edges. Any two-dimensional periodic morphology can be constructed from SSPs in this way, though, as noted above, some morphologies require more than six triangles in an SSP.

**Figure 4:**
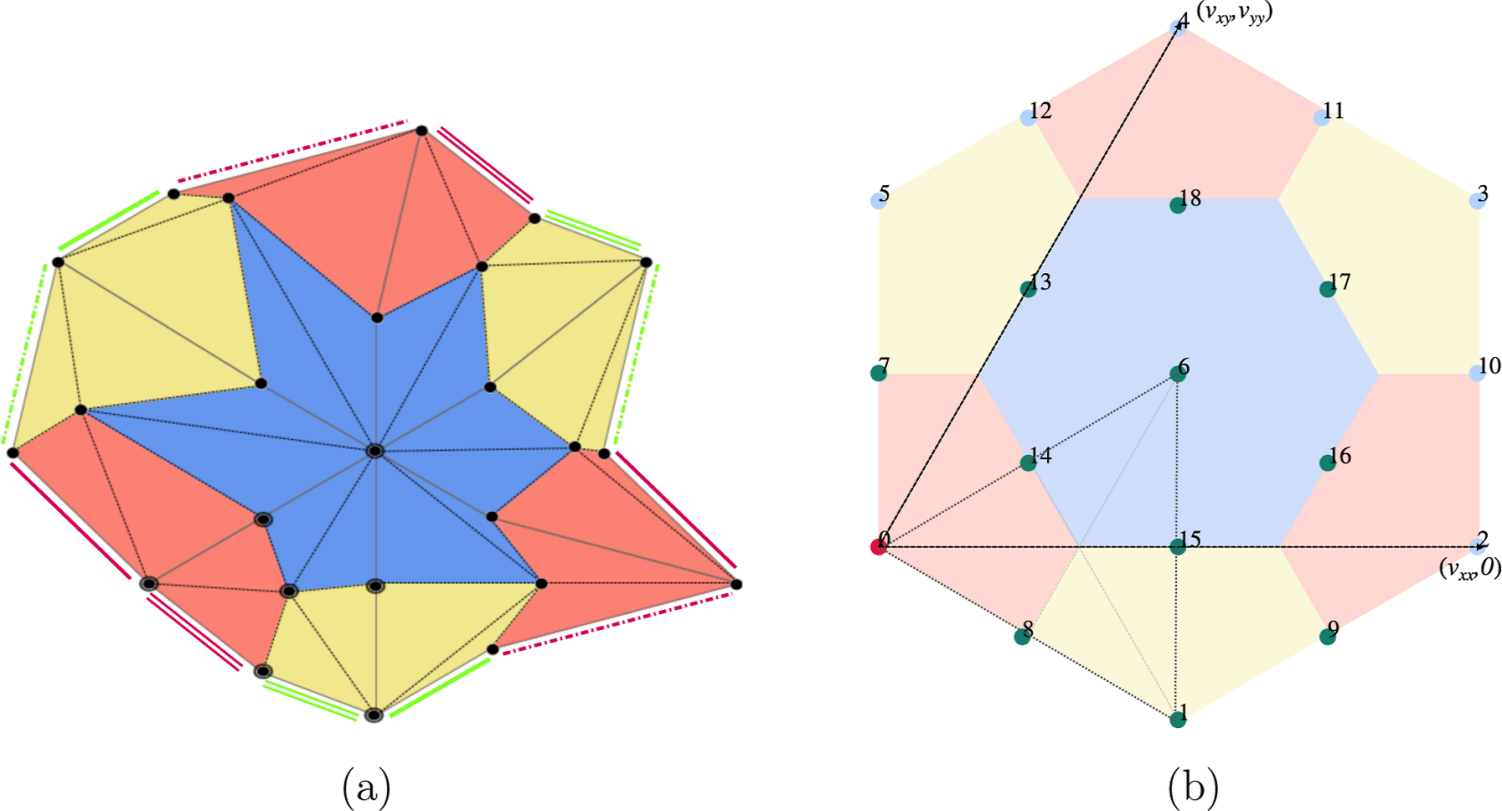
Geometrical structures of SSPs. In (a) we demonstrate how to place six SSPs to form a periodic patch of the [6.6.6] morphology. The black dots indicate the nodes of the SSPs, and one SSP is marked out with larger dots. Colored lines along edges indicate matching rules which makes the morphology periodic. In (a), the same type of lines indicates joining of edges to make the periodic patch. In (b) we give an illustration of the SSP optimization framework. The pink dot is the reference node, green dots are the free nodes and light blue dots are the periodic nodes. The black arrows indicates the periodicity, [*v*_*xx*_, *v*_*xy*_, *v*_*yy*_] of the repeating patch. One SSP is indicated by dashed lines.

Given the locations of all the nodes, the stretching and interfacial energies of each SSP can be computed as described above. The total free energy per chain for the periodic patch is then the sum of free energies of all the SSPs weighted by their areas, and divided by the total area of the patch.

The free energy does not depend on the location of the patch as a whole, so we choose one node, the reference node, to be fixed in space and make it the origin of the coordinate system. This is indicated as a pink dot in Figure 4b. The other nodes are either ‘free nodes’ or ‘periodic nodes’, indicated in green and light blue respectively in Figure 4b. Free nodes can move anywhere in the 2D space, subject to the constraint that all SSPs remain valid (see Supporting Information). Periodic nodes are those nodes on the edge of the patch whose location is constrained by the overall periodicity of the morphology and the locations of the free nodes. The periodicity is specified by choosing two vectors (in two dimensions), (*v*_*xx*_, 0) and (*v*_*xy*_, *v*_*yy*_) as indicated by dotted arrows in Figure 4b. The first vector has a zero *y* component in order to remove rotations, which do not affect the total free energy. The locations of the periodic nodes are derived from the locations of the free nodes using integer sums of these two vectors. For example, the position of a periodic node (*p*_*x*_, *p*_*y*_) is related that of a free node (*x*, *y*) by

12 px=x+lvxx+mvxypy=y+mvyy

where *l* and *m* are integers. Every periodic node is fixed in this way, relative to a particular free node. The choice of assigning nodes as “reference”, “periodic” or “free” is not unique, but the final minimized structure and free energy does not depend on this choice, apart from minor numerical differences that will arise in the minimization procedure.

To take the hexagonal morphology shown in Figure 4b as a specific example, the six vertices labeled 0, 14, 6, 15, 1, and 8 form one SSP, as indicated by gray dotted lines. (The numbering is different from that in Figure 3). Node 0 is the reference node. The linked pairs of free nodes and periodic nodes are [7,10], [0,2], [0,4], [8,11], [1,3], [1,5] and [9,12]. This choice is not unique. The seven nodes in the center are not linked to any other nodes. The configuration of the six SSPs is then specified by the two-dimensional locations of 11 free nodes and the three numbers *v*_*xx*_, *v*_*xy*_ and *v*_*yy*_ that specify the periodicity, making 25 degrees of freedom in all. The free energy of the patch depends on these degrees of freedom and the polymer parameters. Other morphologies are constructed in a similar way.

The total free energy per chain of a patch with a given morphology is minimized in two stages. First, we affinely scale all node positions to find the approximate optimum periodicity lengths for the structure as a whole. Second, we allow all node locations to vary in a manner consistent with the periodicity constraints (while still allowing further small variations in the periodicity). The free energy, viewed as a function of all the degrees of freedom, is minimized using the Broyden–Fletcher–Goldfarb–Shanno (BFGS) algorithm,^58^ as implemented in SciPy.

Using this framework, we compute phase diagrams for the ABC star structure, working out which morphology has the lowest free energy as a function of the composition parameters (ϕ_*A*_, ϕ_*B*_, ϕ_*C*_), for different monomer interaction strengths. While exploring the (ϕ_*A*_, ϕ_*B*_, ϕ_*C*_) phase diagram, for each morphology we usually perform the first minimization with ϕ_*A*_ = ϕ_*B*_ = ϕ_*C*_, starting from the morphology as constructed by hand. For other values of (ϕ_*A*_, ϕ_*B*_, ϕ_*C*_), it is necessary to have a good initial guess for the configuration in order for the minimization to succeed. One cause of the difficulty is that the location of the core is determined by the values of (ϕ_*A*_, ϕ_*B*_, ϕ_*C*_) and the six polygon node locations, and it is possible that the minimization algorithm asks for the free energy for parameter values where the core is outside the SSP, which is invalid. In order to provide good initial guesses, we ‘grow’ the phase diagram from any initial point in (ϕ_*A*_, ϕ_*B*_, ϕ_*C*_), working outward in concentric rings and using neighboring converged configurations as initial guesses, repeating any failed minimizations with alternative nearby initial guesses. This approach is better than the alternative of traversing the phase diagram in multiple linear cuts.

## Results

We will now use the SSP framework to demonstrate the analysis of different morphologies in ABC star terpolymers. We explore both the structure (how the shapes of the domains change) and the stability (which morphology has the lowest free energy) of the phase-separated morphologies. We will examine those single-core and multicore morphologies that have been observed in experiments^24,26,33,39^ and predicted by theoretical work.^16,43^ The ten morphologies we consider in this paper are given in Figure 5 (see also Figure 2). In each case, we create the candidate morphologies by hand from existing patterns using drawing software, identifying the different monomer domains, selecting periodic vertices and calculating vertex locations from the drawing. The spatially periodic region is divided into triangles (SSPs), such that each SSP contains exactly one ABC core. The locations of the vertices of the SSPs and the arrangement of the monomer types define the initial configuration of the SSPs for each morphology.

### Candidate Morphologies

In single-core morphologies, at the minimum free energy of the structure, all SSPs are rotations or reflections of each other throughout the periodic patch. The single-core morphologies that have been observed in experiments are [6.6.6], [8.8.4], [12.6.4] and the lamellar + cylindrical (L + C) morphology (see Table 1). For the [6.6.6] morphology, the periodic patch that we choose is a hexagon, with one domain (for example, B in Figure 5) surrounded by six other alternating domains of the other two monomer types. For the [8.8.4] morphology, the periodic patch is a square, with one domain (A in Figure 5) surrounded by eight alternating domains of the other two monomer types. For the [12.6.4] morphology, the periodic patch is a rhombus. This rhombus is divided into two triangles, each one of them containing one monomer type in the middle (A in Figure 5), surrounded by six alternating domains of the other two monomer types. The final single core morphology we consider is the L + C morphology, with one monomer type forming lamellae (C in Figure 5) with the other two monomer types alternating on either side. Unlike the first three morphologies described here, L + C requires a geometrically asymmetric SSP with eight sides, due to the connectivity of lamellae within this morphology.

In multicore morphologies, the SSPs need not all be the same throughout the patch. The multicore morphologies that we consider are [8.6.4; 8.4.6; 8.6.6], [14.6.4; 14.4.6; 14.4.4], [8.6.4; 8.8.4; 12.6.4; 12.8.4], [10.6.4; 10.4.6; 10.6.6], [10.8.4; 10.6.4] and [12.6.4; 10.8.4; 10.6.4]. These are all considerably more complicated to construct. In practice, after identifying the periodic patch in each case, we identified the locations of all the cores and divided the periodic patch into triangles. We overlaid SSPs onto these triangles, and took the resulting coordinates as our initial configuration. For each structure in Figure 5 we highlight one example of an SSP that corresponds to each of the number triplets in the Schläfli symbols, e.g., for [8.6.4; 8.4.6; 8.6.6], three SSPs are highlighted, corresponding to examples of [8.6.4], [8.4.6] and [8.6.6] cores. Here it is important to note that not all cores with the same number triplets are equivalent by symmetry, and the local environment of their neighboring SSPs may be different. One consequence, which we shall detail below, is that even if two SSPs appear identical in the starting structures of Figure 5, their shape after free energy minimization can be different.

**Figure 5:**
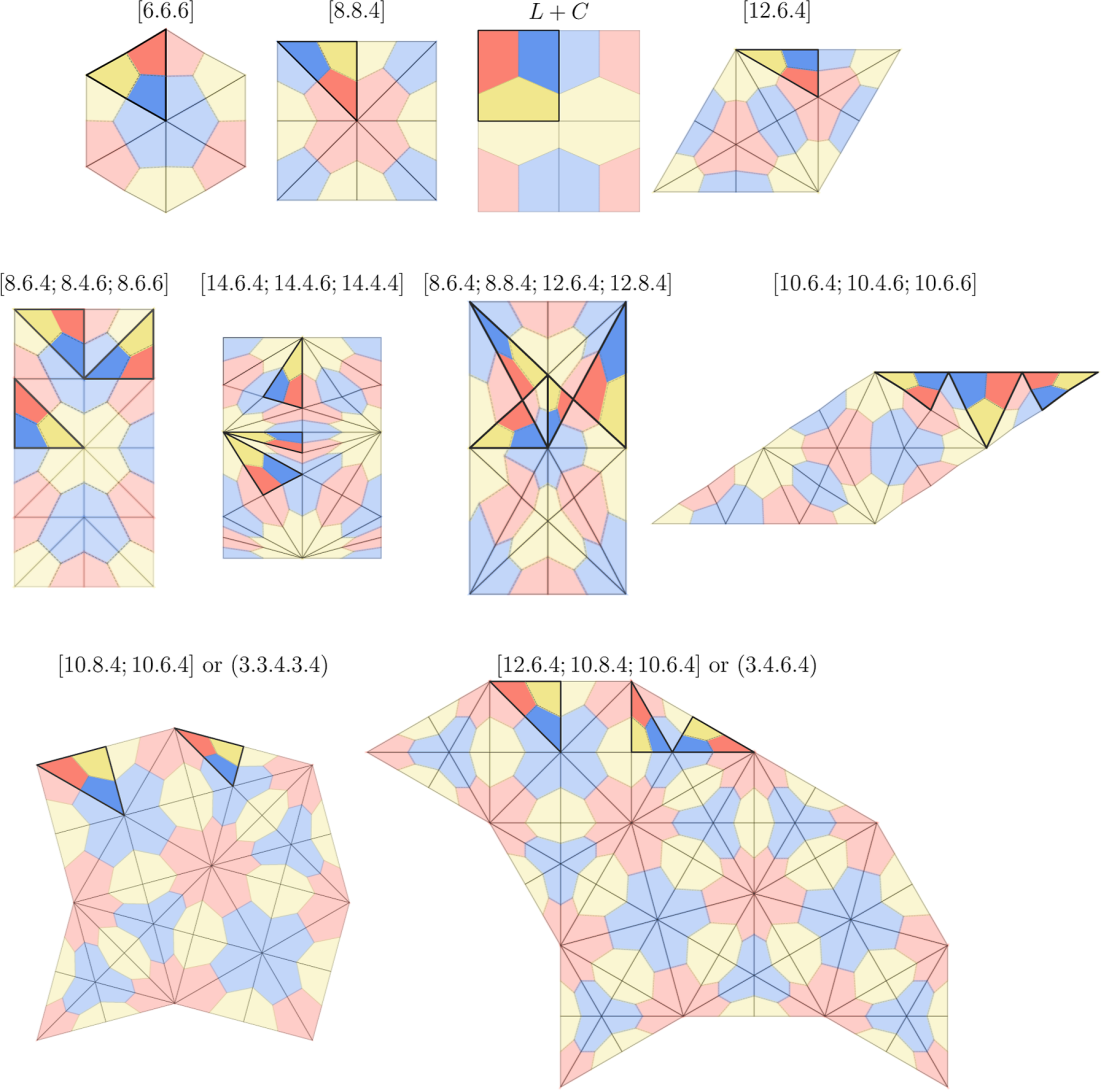
This illustrates how the candidate morphologies considered in this work are discretized into SSPs. The color scheme is *A* (red), *B* (blue) and *C* (yellow). The bold triangles are the SSPs, and each SSP contains six triangles and a single ABC core. In these initial configurations, the SSPs are triangles but there are nodes at the domain boundaries on each edge, so they are in fact six-sided polygons (as in Figure 4). The exception is the L + C example, which has an eight-sided SSP containing eight triangles: two in the red and blue regions and four in the yellow. The SSP polygons are different sizes, but the proportion of red, blue and yellow in each SSP is equal to ϕ_*A*_, ϕ_*B*_ and ϕ_*C*_, which in these examples are all equal to one-third. For each morphology we highlight, with darker shading, a single example of an SSP corresponding to each of the Schläfli symbols. So, for single core-type structures such as [8.8.4] we highlight only one SSP. For multicore structures several SSPs are highlighted, e.g., for [8.6.4; 8.4.6; 8.6.6], three SSPs are highlighted corresponding to examples of [8.6.4], [8.4.6] and [8.6.6] cores. These initial configurations are the starting points for minimizing the free energy.

In addition, each morphology comes in six variants, depending on how the monomer types are allocated to the SSP triangles.

### Construction of Phase Diagrams

We choose values of *N*χ_*AB*_, *N*χ_*BC*_ and *N*χ_*AC*_ and construct ternary phase diagrams with the above-mentioned morphologies with monomer compositions (ϕ_*A*_, ϕ_*B*_, ϕ_*C*_) on each axis. We vary monomer compositions from 0 to 1 with an increment of 0.01, with ϕ_*A*_ + ϕ_*B*_ + ϕ_*C*_ = 1. The free energy minimization struggles when one polymer type dominates, so we restrict to 0.05 ≤ ϕ_*A*_ ≤ 0.90, 0.05 ≤ ϕ_*B*_ ≤ 0.90, 0.05 ≤ ϕ_*C*_ ≤ 0.90, resulting in around 4000 distinct points in the ternary phase diagram. The value of the minimized free energy per chain for each morphology is compared at every point in this diagram, and the morphology with the lowest free energy per chain is marked at that point.

For any given tiling pattern it is possible to produce six different structures by permutation of the monomer “colors” (i.e., red, blue, yellow in our figures). For a given monomer composition and set of interaction parameters, each permutation will have a different free energy. The free energy maps for each of the candidate morphologies are created for all permutations of the domains (ABC, ACB, BAC, BCA, CAB, CBA). The final phase diagram is created by overlaying the free energy maps for all six permutations for the ten candidate morphologies. The morphology with lowest free energy is the one that is marked in the final diagram.

As noted above, the degree of polymerization *N* affects the parameter *c* in the expression for the stretching free energy. This changes the balance between stretching and interfacial energies, and so affects the phase diagram. For most phase diagrams in this paper, we set the degree of polymerization *N* = 1000. We also include phase diagrams with *N* = 300 and *N* = 10, 000 in the Supporting Information and discuss the effect of varying *N* below.

### ABC Star Terpolymers with Symmetric Interactions

The phase diagrams for ABC star terpolymer melts with symmetric interaction strengths (*N*χ_*AB*_ = *N*χ_*BC*_ = *N*χ_*AC*_ = 60) and with *N* = 1000 is given in Figure 6. The value of *N*χ = 60 is chosen in order to be in the strong segregation limit.^18^ This gives $\gamma_{AB}=\gamma_{BC}=\gamma_{AC}=\sqrt{10}$ in eq 9. We vary from this symmetric case in the next section. As expected, the phase diagrams have mirror symmetry in the lines ϕ_*A*_ = ϕ_*B*_, ϕ_*B*_ = ϕ_*C*_ and ϕ_*A*_ = ϕ_*C*_ in the ternary space when we do not distinguish between the color permutations within each morphology.

**Figure 6:**
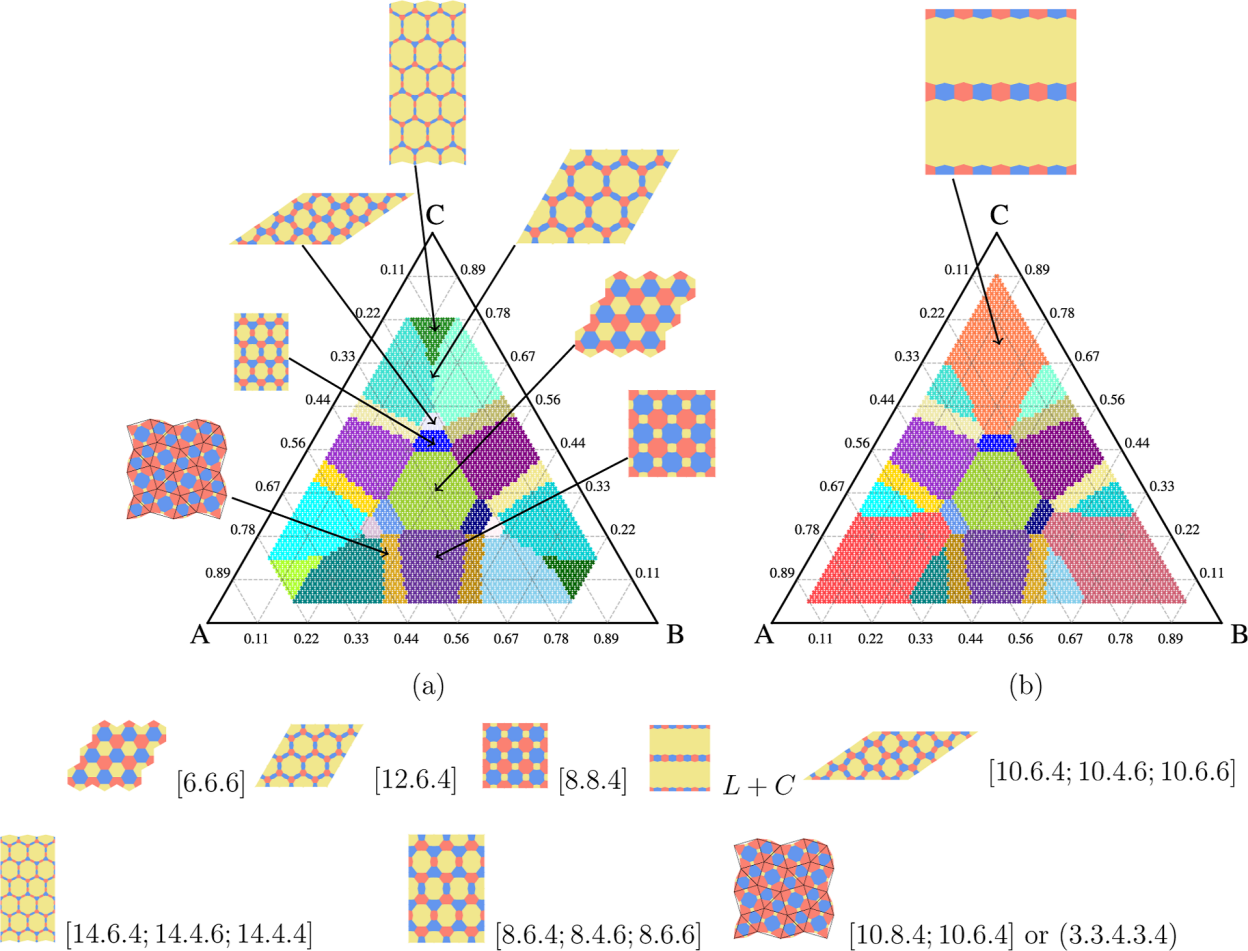
Phase diagrams for ABC star terpolymer with symmetric interaction strengths (*N*χ_*AB*_ = *N*χ_*BC*_ = *N*χ_*AC*_ = 60) and with *N* = 1000. On the left (a) is the phase diagram created using only six-sided SSPs. The stable morphologies are marked around the phase diagram, with the six permutations of each morphology indicated with shades of color. On the right (b) is the phase diagram where we include eight-sided SSPs, which allow lamellar phases. The data for this figure is available from^59^

All of our chosen candidate morphologies in Figure 5 are found in these phase diagrams apart from [8.6.4; 8.8.4; 12.6.4; 12.8.4] and [12.6.4; 10.8.4; 10.6.4]. All morphologies that are experimentally observed in ABC terpolymer systems in Table 1 are present in the phase diagram.

In Figure 6a, we show all morphologies apart from the lamellar phases, and in Figure 6b, we include the lamellar phases as well. These lamellar phases, indicated with orange shades, appear in the corners of the phase diagram. They overtake the entire regions of [10.6.4; 10.4.6; 10.6.6] and [14.6.4; 14.4.6; 14.4.4] phases, and the regions of [12.6.4] are much reduced. The multicore structures that are not obscured are [8.6.4; 8.4.6; 8.6.6] and the Σ-phase. The reason we include both versions of the phase diagram is that in phase diagrams computed using self-consistent field theory,^16^ the lamellar phases are only found in the very corners of the phase diagram. In addition, the Monte Carlo calculations of Gemma et al.^43^ have [12.6.4] phases along the axis of symmetry connecting the corner to the center of the diagram, and again this region is obscured by the lamellar phase. As a result, it is interesting to know what lies underneath the lamellar phases in the central parts of the phase diagram in our strongly segregated calculations. All parts of these diagrams are computed using six-sided SSPs, apart from the lamellar phases in (b), which require eight-sided SSPs.

We first discuss the regions with single-core structures in the phase diagrams in Figure 6. The light green region in the center is where the [6.6.6] morphology has the lowest free energy per chain. This occurs when all three branches have comparable lengths, ϕ_*A*_ ≈ ϕ_*B*_ ≈ ϕ_*C*_. The hexagonal green region where the [6.6.6] morphology has the lowest free energy is bordered by three regions of [8.8.4] (purple shades) and three regions of [8.6.4; 8.4.6; 8.6.6] (blue shades). The [8.8.4] morphology has the lowest free energy when two branches have comparable lengths and the third is smaller than the other two (for example, ϕ_*A*_ ≈ ϕ_*B*_ > ϕ_*C*_). The other single-core structures ([12.6.4]) are indicated in teal/light blue shades, and they occupy the corner regions of the ternary space, where all three chains have different lengths, with one chain significantly longer than the other two (for example, ϕ_*A*_ > 0.5 > ϕ_*B*_ > ϕ_*C*_). It is interesting to observe that single-core structures occupy the majority of the phase diagram.

Next we turn to regions with multicore structures, which are stable at the intersections of the [6.6.6], [8.8.4] and [12.6.4] single-core regions. Between the [6.6.6] region in the center and the six outer [12.6.4] regions, we find (moving outward) [8.6.4; 8.4.6; 8.6.6] (trapezoidal shaped regions indicated with blue shades) and [10.6.4; 10.4.6; 10.6.6] (triangular shaped regions indicated with lavender shades). In all these regions, the compositions of two of the polymer types are comparable, while the third is somewhat longer than the other two: none of the three branches are extremely small. Toward the corners of the phase diagram, and at the intersection of regions occupied by two topological permutations of [12.6.4], there are regions of [14.6.4; 14.4.6; 14.4.4] (indicated with shades of dark green). Like the [12.6.4] structures, this morphology has a large number of cores surrounding one central domain, and in these regions, the compositions of two of the polymer types are small and comparable, while the third is considerably longer than the other two.

The third type of multicore structure that we find is the [10.8.4; 10.6.4], also known as the Σ-phase and (3.3.4.3.4). The six rectangular-shaped regions of this phase (indicated with yellow shades) are found between the (purple) [8.8.4] and (teal) [12.6.4] regions. The [8.8.4] phase comprises square tiles (made from joining the centers of the red, blue or yellow domains), and the [12.6.4] phase comprises triangular tiles (made from joining the centers of the largest of the three colors). Since the Σ-phase is a combination of square tiles and triangular tiles, it is natural to find this phase between the regions of [8.8.4] and [12.6.4]. In the phase diagram given in Figure 6a, all the rectangular regions corresponding to different topological subclasses of the Σ-phase occupy equal areas. In these calculations using symmetric interactions, we find the Σ-phase at roughly the same position in the phase diagram (using our SSP method) as reported by Ueda et al.^46^ (using Monte Carlo methods) and by Li et al.,^18^ Xu et al.^51^ and Cody et al.^16^ (using SCFT). While the rough locations of the regions of Σ-phase are similar, our regions are somewhat larger than those reported in the literature.

In the Supporting Information, we show phase diagrams for *N* = 300 and *N* = 10, 000. These are qualitatively similar to the *N* = 1000 phase diagrams presented here. The most important effect of changing *N* is to change the extent of the L + C phases. With smaller *N* = 300, the regions occupied by the L + C phases are larger, and as a consequence, the regions occupied by [12.6.4] are reduced. With larger *N* = 10, 000, the regions occupied by the L + C phases are smaller, revealing the previously hidden [10.6.4; 10.4.6; 10.6.6] structures. There are also small changes in the positions of the boundaries between other phases, but without a qualitative change to the overall phase diagram.

The phase diagrams shown in Figure 6 agree with ternary phase diagrams for ABC terpolymer systems reported by Hawthorne et al.,^60^ Li et al.,^18^ and Zhang et al.,^50^ which were obtained using SCFT for 2D morphologies. Overall, the types of morphologies observed are qualitatively similar; however, a notable difference arises for the L + C phase. In prior studies, the L + C and other lamellar phases occupy relatively limited regions of the phase space, whereas in our phase diagram (Figure 6(b)) L + C occupies a substantial portion of the diagram. Multicore morphologies, [10.6.4; 10.4.6; 10.6.6] and (3.4.6.4), which are reported in earlier work, are absent in our results. As *N* increases, some multicore morphologies reappear, and the resulting phase diagrams progressively resembles those predicted by SCFT.

### ABC Star Terpolymers with Asymmetric Interactions

The SSP technique allows us to vary parameters with relative ease and so we can readily study morphologies with asymmetric interaction strengths. We start from the symmetric phase diagrams in Figure 6, with (*N*χ_*AB*_, *N*χ_*BC*_, *N*χ_*AC*_) = (60, 60, 60) with *N* = 1000, and recalculate all minimized free energies with different values of (*N*χ_*AB*_, *N*χ_*BC*_, *N*χ_*AC*_).

In Figure 7, we report three cases of different asymmetric interactions. The three cases are

1. *N*χ_*AB*_ = *N*χ_*BC*_ = 120, *N*χ_*AC*_ = 60, so there is less repulsion between *A* and *C* monomers compared to the repulsion between the other two pairs, which are equal.
2. *N*χ_*AB*_ = *N*χ_*BC*_ = 60, *N*χ_*AC*_ = 120, so there is more repulsion between *A* and *C* monomers compared to the repulsion between the other two pairs, which are equal.
3. *N*χ_*AB*_ = 120, *N*χ_*BC*_ = 60, *N*χ_*AC*_ = 180, so *B* and *C* repel each other less strongly, and *A* and *C* repel each other more strongly, compared to the interaction between *A* and *B*.

**Figure 7:**
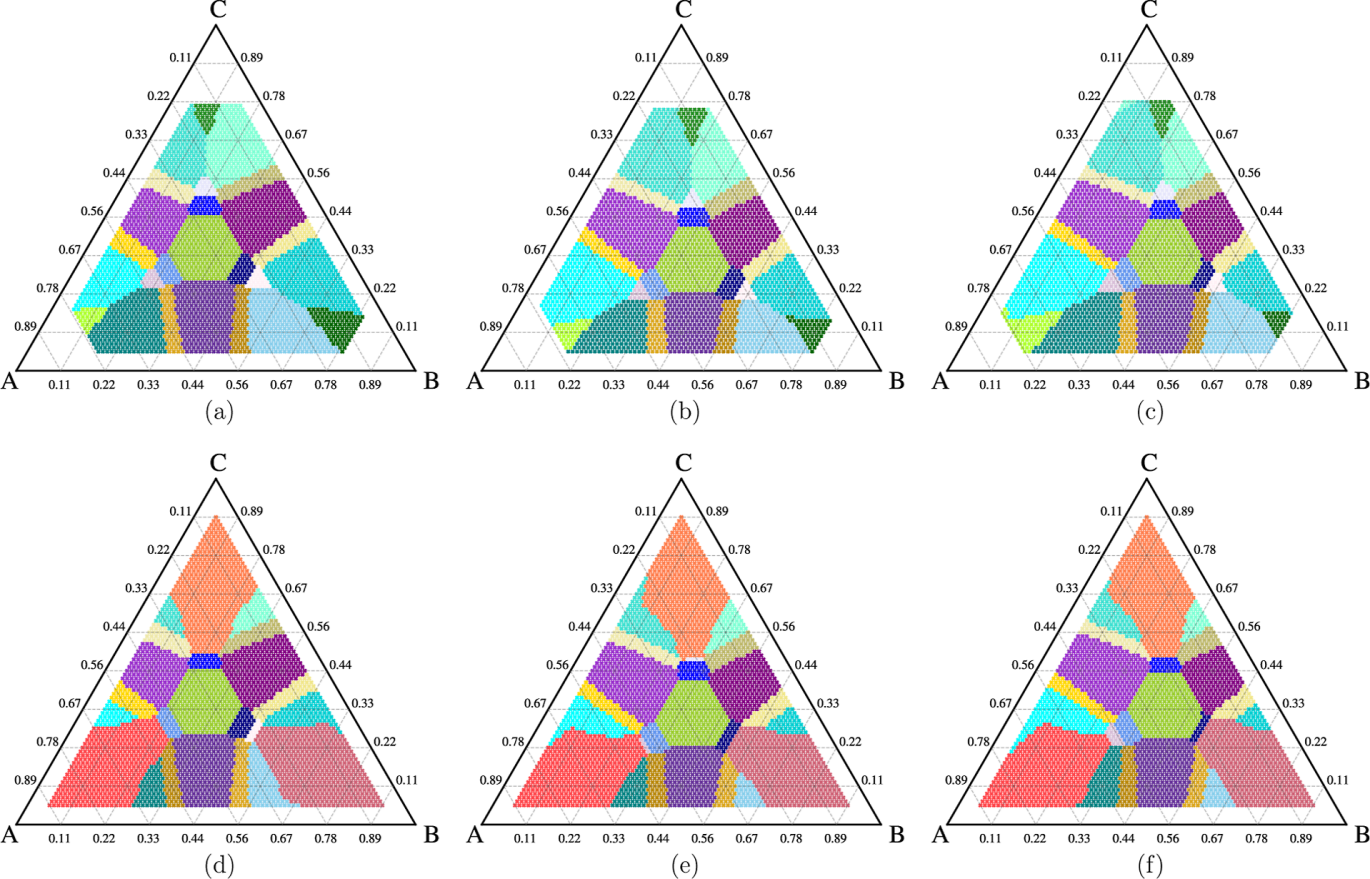
Phase diagrams of ABC star terpolymers with asymmetric interactions and with *N* = 1000. In (a,d), *N*χ_*AB*_ = *N*χ_*BC*_ = 120, *N*χ_*AC*_ = 60. In (b,e), *N*χ_*AB*_ = *N*χ_*BC*_ = 60, *N*χ_*AC*_ = 120. In (c,f), *N*χ_*AB*_ = 120, *N*χ_*BC*_ = 60, *N*χ_*AC*_ = 180. The top row has phase diagrams with morphologies using only six-sided SSPs. The bottom row has phase diagrams that include lamellar morphologies, constructed using eight-sided SSPs. The colors indicating the morphologies are same as in the symmetric phase diagrams in Figure 6. The data for this figure is available from^59^

Phase diagrams for these three cases using the six-sided SSPs with *N* = 1000 are given in Figure 7a–c, and the phase diagrams including eight-sided SSPs are given in Figure 7d–f.

As expected, we observe shifts in the phase diagrams relative to the symmetric case. For the cases 1 and 2, given in Figure 7a,d and Figure 7b,e, there is a mirror symmetry with respect to the diagonal line ϕ_*A*_ = ϕ_*C*_, as expected from having *N*χ_*AB*_ = *N*χ_*BC*_. This symmetry is not present when all interactions are different. The overall phase diagram is similar to the symmetric case, with same relative positions of the different regions in the phase diagram, but the sizes of the regions change and there is an overall shift. In the case with *N*χ_*AB*_ = *N*χ_*BC*_, this shift is along the line of symmetry (ϕ_*A*_ = ϕ_*C*_), with all regions moving away from the B corner when *N*χ_*AC*_ < *N*χ_*AB*_ and moving toward the B corner when *N*χ_*AC*_ > *N*χ_*AB*_.

With changing the values of the *N*χ parameters, we observe that regions occupied by different topological subclasses within the same morphology are no longer identical. For example, in Figure 7a,d, the yellow regions corresponding to the stable Σ-phase and located farther from the B corner are wider than the other four regions. In contrast, in Figure 7b,e and c,f, the yellow regions farther from the B corner are thinner than the other four stable Σ-phase regions in their respective phase diagrams.

The [10.6.4; 10.4.6; 10.6.6] regions (lavender shades) were masked by the lamellar phases (orange shades) in the symmetric case, but with asymmetric interactions, decreasing *N*χ_*AC*_ reveals those regions with larger *B* domains, and vice versa. In the more extreme example in Figure 7f, the [10.6.4; 10.4.6; 10.6.6] region closest to the A corner is partially revealed with the small value of *N*χ_*BC*_, while the [8.6.4; 8.4.6; 8.6.6] region (blue shades) closest the B corner (as well as the [10.6.4; 10.4.6; 10.6.6] region) is completely masked by the lamellar phase.

### Structural Analysis of Minimized Morphologies

In the existing literature on polymer phase separation, there is little discussion of the shape of local monomer domains and the curvature of their interfaces. Our SSP method allows us to visualize the domains and investigate the interfaces between them, both at the scale of the local monomer domains and at the scale of the tiles that make up the morphology.

We first examine the shapes of SSPs in the multicore structures. As noted above, within each structure, SSPs with the same Schläfli symbol number triplets are not necessarily equivalent by symmetry, having different local environments. As illustrated in Figure 8, this affects their area and shape in the structures after free energy minimization. For the [8.6.4; 8.4.6; 8.6.6] morphology shown in Figure 8a, all [8.6.4] SSPs are symmetrically equivalent and so take the same shape upon minimization; likewise for the [8.4.6] SSPs. However, the [8.6.6] SSPs fall into two classes, marked as [8.6.6]* (with a concave red–yellow edge) and [8.6.6]** (with a concave blue–yellow edge) in the figure. Similarly, for the Σ-phase in Figure 8b, there are two classes of [10.8.4] and three classes of [10.6.4] SSPs, each with different shapes in the minimized structure.

**Figure 8:**
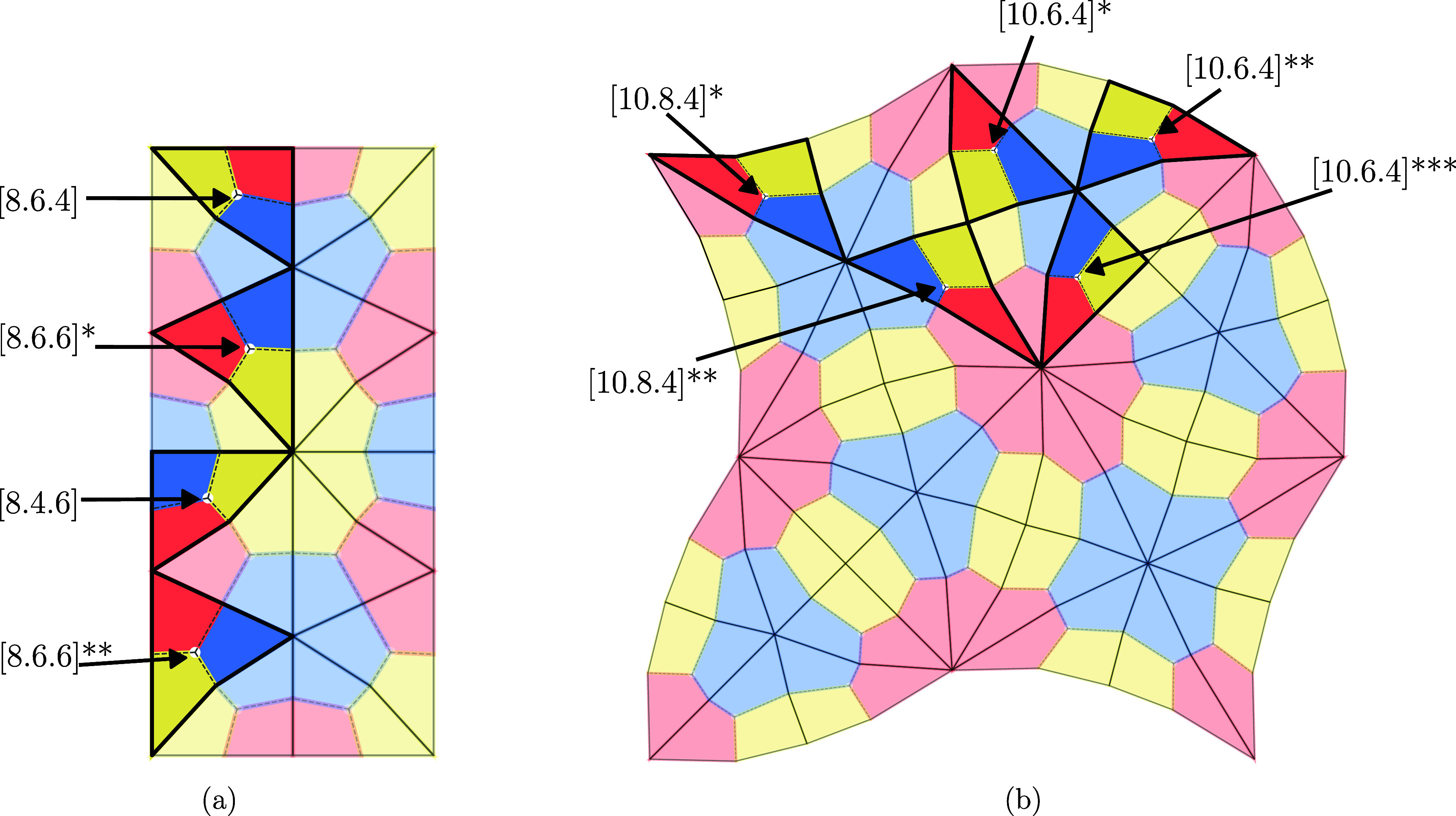
Periodic patches of morphologies [8.6.4; 8.4.6; 8.6.6] and Σ-phase after minimization are shown with highlighted SSPs. Examples of each symmetrically distinguishable SSPs are highlighted. In (a), [8.4.6] and [8.6.4] are highlighted as well as two types of [8.6.6]. In (b), two types of [10.8.4] and three types of [10.6.4] are highlighted.

As we noted in the introduction, it is helpful for construction of the morphology to view the Σ-phase as being composed of square and triangular tiles. At the level of these tile shapes, the initial configuration of the Σ-phase in Figure 5 contains two square and four equilateral triangle tiles, all with with straight edges. Upon minimization, we obtain the morphology state given in Figure 9a. There are significant changes in the tile shapes: all edges of the square tiles curve inward, and two edges of every triangle tile curve outward. In Figure 9a, the original (unminimized) tile edges are straight black lines, and the tile edges after minimization are shown as dashed white lines. The edges between two adjacent triangles are straight, while the edges between triangle and square tiles curve toward the square tile. This is seen most clearly in the locations of the yellow domains, which are centered on the edges between two triangles, but are shifted toward the squares when they are between a triangle and a square. We observe the same effect for all value of the monomer compositions and interaction strengths, and for all morphologies that involve both square and triangle tiles.

**Figure 9:**
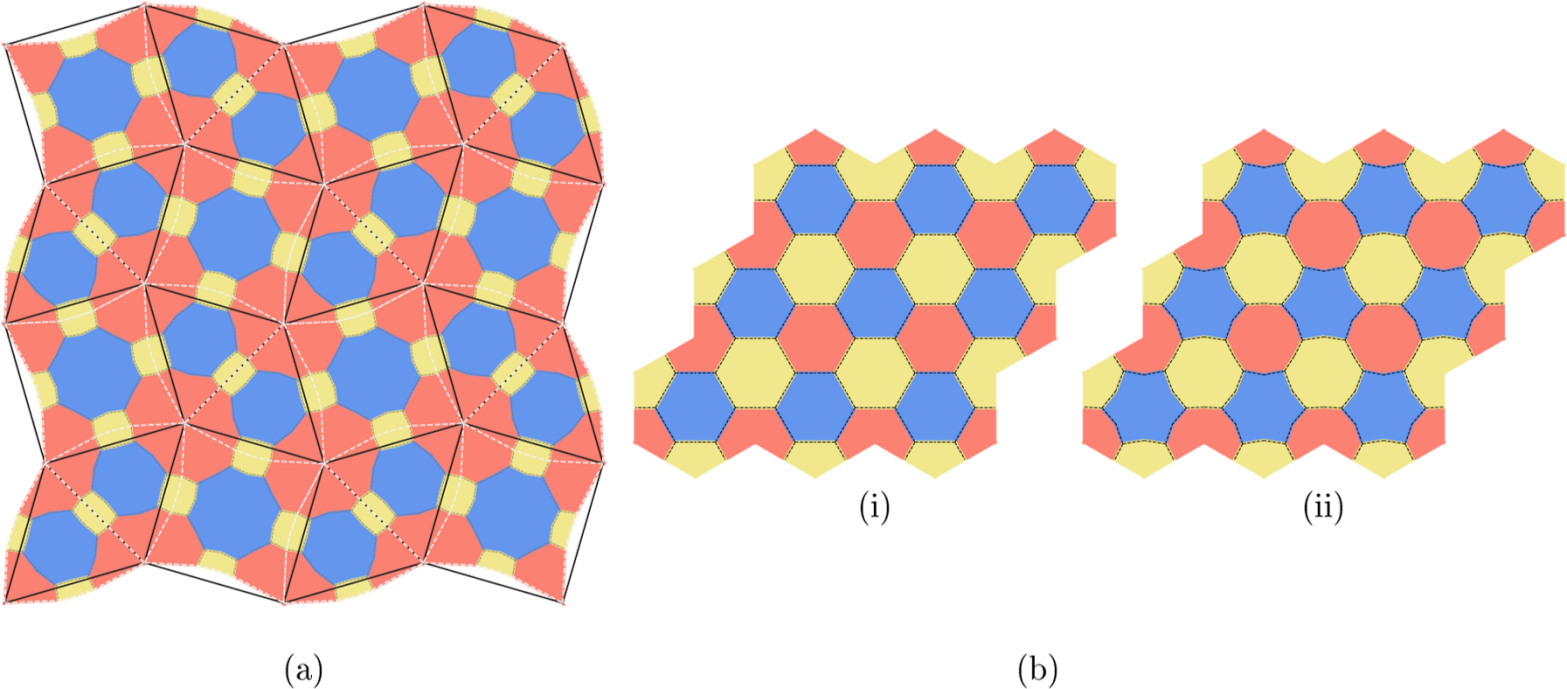
Minimized structure for (a) the Σ-phase and (b) [6.6.6] with different interaction strengths. In (a), we have ϕ_*A*_ = 0.57 (red), ϕ_*B*_ = 0.38 (blue), ϕ_*C*_ = 0.05 (yellow) and *N*χ_*AB*_ = *N*χ_*BC*_ = *N*χ_*AC*_ = 60. The original (unminimized) tile edges are straight black lines, and the tile edges after minimization are shown as dashed white lines In (b), we have $\phi_{A}=\phi_{B}=\phi_{C}=\frac{1}{3}$ , and in (i), *N*χ_*AB*_ = *N*χ_*BC*_ = *N*χ_*AC*_ = 60, while in (ii), *N*χ_*AB*_ = 120, *N*χ_*BC*_ = 60 and *N*χ_*AC*_ = 180.

Zeng et al.^61^ attribute the changes of the tile shapes (in a related system) to the difference in the number of core per unit area between the tiles: with edges of unit length, squares (with 8 cores) have 8 cores per unit area, while triangles (with 6 cores) have $24/\sqrt{3}\approx 13.9$ cores per unit area. Balancing the stretching energies between these two requires squares to shrink and triangles to expand. This effect is seen in SCFT calculations^16,18^ and in the experimental results reported by Hayashida et al.,^35^ but was not remarked.

At the level of the local monomer domains, our SSP method provides information about the shapes of the interfaces between these domains. In Figure 9b, we show how changing the interaction strengths affects the shapes of the domains. In (i), with $\phi_{A}=\phi_{B}=\phi_{C}=\frac{1}{3}$ and *N*χ_*AB*_ = *N*χ_*BC*_ = *N*χ_*AC*_ = 60, the monomers are all equivalent, and the morphology with the lowest free energy is perfect hexagons, with all monomer domains having the same area. In (ii), we change the interaction strengths and set *N*χ_*AB*_ = 120, *N*χ_*BC*_ = 60 and *N*χ_*AC*_ = 180, resulting in curved interfaces while keeping all the areas the same. Since *N*χ_*AC*_ is the largest, the incompatibility between *A* and *C* domains is minimized by reducing the length of the AC (red–yellow) interfaces. Similarly, *N*χ_*BC*_ is the smallest, resulting in longer *BC* (blue–yellow) interfaces. The corners of the *A* domains are all attached to *BC* interfaces, which can move most easily, resulting more circular *A* domains, with *AB* and *AC* interfaces curving outward from the *A* domain. Conversely, the corners of *B* domains are pulled by the shorter *AC* interfaces, resulting in pointed corners in *B* domains, with *AB* and *BC* interfaces curving into the *B* domain.

We illustrated this effect with the equal-area [6.6.6] morphology, but similar arguments apply to other morphologies. Higher interactions (higher surface tensions) tend to favor more circular domains. This general principle helps to rationalize the shifts in the phase diagram observed above in Figure 7. Domains that have higher numbers of neighbors are able more easily to approximate a circle. Hence, in regions of the phase diagram where two morphologies are competing, higher interaction strengths for a given monomer type will tend to favor morphologies where the number of neighbors is larger for domains of those monomers. This can be observed, for example, in Figure 7a, where *N*χ_*AB*_ = *N*χ_*BC*_ = 120, *N*χ_*AC*_ = 60, so that the interactions involving the B monomers are largest. Here one can observe that the [12.6.4] phases with 12-neighbor *B* domains displace the [10.8.4; 10.6.4] Σ-phases with 10-neighbor B domains, which in turn displace the [8.8.4] phases with 8-neighbor *B* domains. These in turn displace the [6.6.6] phase, which (finally) displaces the [8.8.4] phases with 4-neighbor *B* domains. In every case, the morphology where the *B*-domain has the larger number of neighbors “wins”. The net effect is that the overall phase diagram appears to shift away from the B corner toward the A-C boundary. An exception to this shifting trend is the L + C morphology. For example, when *B*-domains are lamellar they cannot approximate a circle, and so this morphology does not increasingly outcompete other phases when the *B* interactions are increased. Hence, while other phases shift in the phase diagram, the L + C phase does not, which is why we see the emergence of the [10.6.4; 10.4.6; 10.6.6] phase toward the bottom right of Figure 7d as it outcompetes the L + C phase on the one side and [8.6.4; 8.4.6; 8.6.6] on the other.

## Conclusions

We have developed a method for investigating different morphologies in ABC star terpolymers in the strong segregation limit. Our method is based on surrounding each ABC core with a polygon, and then adjusting the locations of the vertices of all the polygons to find an overall minimum of the free energy. We mainly use six-sided polygons, and eight-sided polygons for certain morphologies, but the method can be extended to polygons with more sides if needed (for example, if greater resolution of interfacial shapes was required). This improves on the method of Gemma,^43^ who did strong segregation calculations of ABC star terpolymers using triangles rather than polygons around each core. Other strong segregation calculations of AB diblock^2,15^ and ABC triblock^62^ primarily used wedges or other elements to parametrize the shapes of the regions. For ABC star terpolymers, our polygons are the simplest way of describing the phase separated structures, and so our work is a significant addition to the strong segregation theory toolbox. We have considered here only two-dimensional tiling-based periodic structures; in principle, the method can be extended to consider periodic approximants to aperiodic (quasicrystalline) structures.

Apart from the work of Gemma,^43^ there are no other strong segregation calculations for these kinds of ABC star structures in the literature, though there are many self-consistent field theory calculations of these structures and more. Strong segregation theory calculations are much more straightforward than self-consistent field theory and other mean field theory calculations. Using strong segregation theory allows rapid exploration of many different structures in the phase diagram. Using strong segregation theory, combined with our approach of ‘growing’ phase diagrams from a converged structure, enables us to compute entire phase diagrams with much less computational effort than alternative methodologies (self-consistent field theory, dissipative particle dynamics and Monte Carlo).

In particular, we have computed phase diagrams for all the known two-dimensional structures in ABC star terpolymer systems, with both equal and unequal Flory interaction parameters, considering a wider range of asymmetric variations than elsewhere in the literature. We observe that single core morphologies ([6.6.6], [8.8.4], [12.6.4] and L + C) occupy the majority of all the phase diagrams, with the multicore Σ-phase and [8.6.4; 8.4.6; 8.6.6] also present. While we focused on the *N* = 1000 case, our *N* = 300 and *N* = 10, 000 phase diagrams in the Supporting Information show that the regions of L + C are reduced and the multicore [10.6.4; 10.4.6; 10.6.6] morphology that was masked by L + C can be revealed with larger *N*. This limit is appropriate for SCFT calculations, which find the same multicore structure and a reduced prominence for lamellar phases. Experiments with pure ABC star terpolymers record only single-core structures (see Table 1, where structures featuring lamellae are prominent). With unequal interaction parameters, we observe a shift in the phase diagram consistent with the self-consistent field theory calculations of Jiang et al.^53^ This shift in the phase diagram also allows the multicore [10.6.4; 10.4.6; 10.6.6] morphology to be revealed from behind the L + C morphology. More widely, similar shifts in phase diagrams are observed in diblock and other systems once asymmetry is introduced, which can reveal more complex phases such as Frank–Kasper phases.^15^

We were able to rationalize the shifts in the phase diagram with varying interaction parameters by noting that higher interactions gives higher interfacial tension and so a preference for more circular domains with a larger number of neighbors. The dependence of phase diagrams on *N* (independently of the product *N*χ) arises because of the logarithmic dependence of the stretching energy on the core radius *R*_core_ in eq 1. Thus, changing *N* affects the balance between stretching and interfacial energies, giving small shifts in the phase boundaries.

The method can be extended in a variety of ways. For instance, the effects of conformational asymmetry can be introduced by allowing the step length parameter *b* to be different for the different monomer types. The method could be used to investigate mixtures of more than one type of terpolymer, for example, mixtures of ABC and ABD terpolymers, or mixtures of ABC terpolymer and A homopolymer (as used experimentally by Hayashida et al.^35^).

Other potential extensions to the method involve modifications to the SSP structure. In this paper we have used SSPs with a minimal number of nodes to represent the morphologies we have investigated, using six-sided SSPs for the majority of the morphologies and eight-sided SSPs for the L + C phase. Adding extra nodes would permit a more refined calculation of both structure and free energy, at the computational expense of an increased number of degrees of freedom. Extra nodes along the edges of the SSP would permit a higher-order representation of the boundaries between SSPs where these are not straight, which occurs only in the multicore structures as illustrated in Figure 8. One could also consider adding nodes to obtain a higher-order approximation of the lines from the central node to the exterior of the SSP in Figure 3, allowing a better representation of curved interfaces between domains and, correspondingly, nonstraight wedges along which the chains stretch.

We have only implemented the method in two dimensions, though in principle it could be extended to consider three-dimensional structures. This would naturally involve extending each SSP into the third dimension, so that they comprise two hexagonal faces (each essentially identical to our 2D SSPs) connected by lines joining the equivalent outer and central nodes from the two faces. For maximal flexibility, the triangles within the hexagonal faces need not be coplanar in 3D, and nor do the two hexagonal faces need to be parallel. By stacking together a number of such base motifs, it will possible to approximate effects that arise in three-dimensional structures, such as core lines that are not straight. However, almost all ABC star terpolymer structures that have been reported experimentally are two-dimensional (see Table 1).

We also believe that it is possible to construct similar tessellating base motifs to perform SST calculations for other copolymer architectures. As noted in the Introduction for example, Reddy, Dimitriyev and Grason^4,5^ constructed a strong segregation theory for bicontinuous phases of AB copolymers by making use of “wedges”,^2^ which are the tessellating base motif structure for an AB copolymer, equivalent to our SSPs.

In future, we plan to use this method to investigate quasicrystal approximants in ABC star terpolymers, inspired by the tilings presented by Wang et al.^16^ The efficiency of the method will allow us to reach reasonably large approximants.

## Supplementary Material



## Notes

In the Supporting Information, we give details of how we determine the interfacial energy and stretching free energy per chain in a Strongly Segregated Polygon (SSP). We also give phase diagrams in the *N* = 300 and *N* = 10, 000 cases (PDF)
