# Supplementary material for: A Flexible Implementation of Strong Segregation Theory for Two-Dimensional ABC Star Terpolymer Morphologies
Source: Macromolecules. 2026 Jun 15;59(13):7663–78. doi: 10.1021/acs.macromol.5c03113 (PMC13374385; doi:10.1021/acs.macromol.5c03113)
Supplement: Supplementary file 1 [file ma5c03113_si_001.pdf]

A flexible implementation of strong segregation theory for two dimensional ABC star terpolymer morphologies: Supporting Information

Merin Joseph,*,†,‡ Daniel J. Read,*,¶ and Alastair M. Rucklidge*,¶

†*Department of Chemistry, Technical University of Denmark, Kgs. Lyngby, Denmark*

‡*Niels Bohr Institute, University of Copenhagen, Denmark*

¶ *School of Mathematics, University of Leeds, Leeds LS2 9JT, UK*

E-mail: merin.joseph@nbi.ku.dk; D.J.Read@leeds.ac.uk; A.M.Rucklidge@leeds.ac.uk

In this Supporting Information, we will give details of how we determine the interfacial energy and stretching free energy per chain in a Strongly Segregated Polygon (SSP). We also give phase diagrams in the $N = 300$ and $N = 10,000$ cases.

Free energy calculation of Strongly Segregated Polygons

The six-sided SSP is the basic unit in our method, and an example is given in fig. S1. The polygon is divided into three regions as indicated by different colors in fig. S1, where each region is assigned to different monomer types. The color map is red for A, blue for B and yellow for C. The six nodes on the perimeter are defined by their x and y coordinates. The position of the central node, which is the core of the ABC star terpolymer, follows from these positions and from the area fractions of A, B and C, as explained below. The central

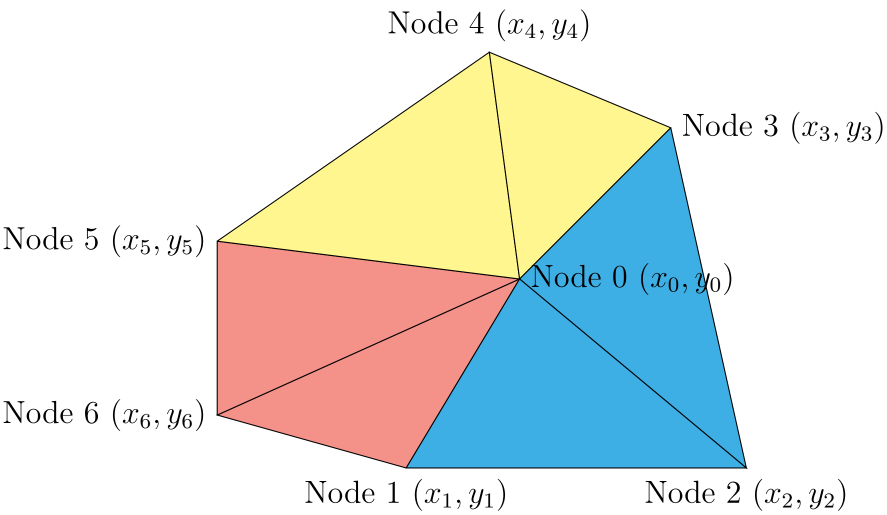

Figure S1: The structure of an SSP with seven nodes. All the nodes Node i and their position coordinates are given. We assign red, blue and yellow colors to domains of A, B and C monomer types.

node needs to be inside the polygon for it to be valid. Subject to this constraint, the six perimeter nodes have freedom to move around anywhere.

The SSP is divided into six triangles: 012, 023, 034, 045, 056 and 061, where node 0 is the central node and nodes 1 to 6 are counterclockwise (in this case) around the perimeter. In fig. S1, the interfaces are line segments between nodes 0 and 1 (between polymers A and B), between nodes 0 and 3 (between B and C) and between nodes 0 and 5 (between C and A). The contributions from these are added up to give the interfacial energy for the SSP. The stretching free energy per chain can be computed for each triangle and added up. Thus the free energy per chain for the ABC stars within the SSP is determined uniquely by the positions of the nodes, and the free energy minimization can be performed by adjusting the node positions.

In order to determine the position of node 0, we relate the monomer compositions to the

areas covered by the three types of triangle as follows:

$$\begin{aligned} \phi_A &= \frac{A_{061} + A_{056}}{A_T}, \\ \phi_B &= \frac{A_{012} + A_{023}}{A_T}, \\ \phi_C &= \frac{A_{034} + A_{045}}{A_T}, \end{aligned} \tag{S1}$$

where A_T is the total area of the SSP. The area of each triangle is expressed in terms of the node coordinates in vector form. The position vector of node i with respect to the core (node 0) is $\vec{r}_{0i}$. Following this labelling convention, the signed (vector) areas are:

$$\begin{aligned} \vec{A}_{061} &= \frac{1}{2}(\vec{r}_{06} \times \vec{r}_{01}); \quad & \vec{A}_{012} &= \frac{1}{2}(\vec{r}_{01} \times \vec{r}_{02}); \\ \vec{A}_{023} &= \frac{1}{2}(\vec{r}_{02} \times \vec{r}_{03}); \quad & \vec{A}_{034} &= \frac{1}{2}(\vec{r}_{03} \times \vec{r}_{04}); \\ \vec{A}_{045} &= \frac{1}{2}(\vec{r}_{04} \times \vec{r}_{05}); \quad & \vec{A}_{056} &= \frac{1}{2}(\vec{r}_{05} \times \vec{r}_{06}). \end{aligned} \tag{S2}$$

We use the signs of these areas to check the validity of the polygon: any polygon in which some areas are positive and some are negative is invalid, since this would imply that the triangles overlap. This statement is equivalent to the requirement that the core (node 0) should be inside the SSP. In calculations, we use positive areas, so $A_{061} = \left|\vec{A}_{061}\right|$, and so on. The total unsigned area A_T is the sum of the six triangle areas.

The three equations in eq. (S1), with the incompressibility constraint $\phi_A + \phi_B + \phi_C = 1$, can be solved for (x_0, y_0) to determine the position of the core as a function of the monomer compositions and the known coordinates of other six nodes. On solving, the position of the

core is

$$
\begin{aligned}
x_0 = {} & \frac{\left(2(1-\phi_A-\phi_B)A_T - x_3y_4 - x_4y_5 + x_4y_3 + x_5y_4\right)(x_1-x_5)}{(y_3-y_5)(x_1-x_5)-(y_5-y_1)(x_5-x_3)} - \\
& \frac{\left(2\phi_A A_T + x_6y_5 + x_1y_6 - x_5y_6 - x_6y_1\right)(x_5-x_3)}{(y_3-y_5)(x_1-x_5)-(y_5-y_1)(x_5-x_3)}, \\
y_0 = {} & \frac{\left(2(1-\phi_A-\phi_B)A_T - x_3y_4 - x_4y_5 + x_4y_3 + x_5y_4\right)(y_1-y_5)}{(y_3-y_5)(x_1-x_5)-(y_5-y_1)(x_5-x_3)} - \\
& \frac{\left(2\phi_A A_T + x_6y_5 + x_1y_6 - x_5y_6 - x_6y_1\right)(y_5-y_3)}{(y_3-y_5)(x_1-x_5)-(y_5-y_1)(x_5-x_3)}.
\end{aligned}
\tag{S3}
$$

The denominator in these expressions is zero only when Nodes 1, 3 and 5 are co-linear, which corresponds to the (physically impossible) situation where the internal AB, AC and BC interfaces are co-linear. The calculation for the eight-sided SSPs is similar.

Interfacial energy in an SSP

In the SSP, the three interface lengths are l_{01}, l_{03} and l_{05}, the lengths between node 0 and nodes 1, 3 and 5, which have not (yet) been scaled by the length $R = \sqrt{Nb^2}$. The polygon extends along the unscaled length d of the core cylinder in the third dimension. The interfacial energy at each surface IJ (between chains of type I and J) will be the surface tension $\tilde{\gamma}_{IJ}$ times the area of that surface, where $\tilde{\gamma}$ is units of k_BT per unit area. The total interfacial energy, in units of k_BT, is

$$
F_{int} = \tilde{\gamma}_{AB}\tilde{A}_{AB} + \tilde{\gamma}_{BC}\tilde{A}_{BC} + \tilde{\gamma}_{AC}\tilde{A}_{AC}, \tag{S4}
$$

where $\tilde{A}_{AB} = dl_{01}$, $\tilde{A}_{BC} = dl_{03}$ and $\tilde{A}_{AC} = dl_{05}$ are the interfacial areas. The number of ABC star terpolymer chains in the volume is $\tilde{A}_T d/v_p$, where v_p is the total volume of each terpolymer chain. Thus, the interfacial energy per chain f_{int}, in units of k_BT, is

$$
f_{int} = (\tilde{\gamma}_{AB}l_{01}d + \tilde{\gamma}_{BC}l_{03}d + \tilde{\gamma}_{AC}l_{05}d) \times \frac{v_p}{\tilde{A}_T d}. \tag{S5}
$$

We recall the relationship between the surface tension $\tilde{\gamma}$ and the Flory interaction parameter χ:

$$\tilde{\gamma}_{IJ} = \sqrt{\frac{\chi_{IJ}}{6}}\rho b, \tag{S6}$$

where $\rho = N/v_p$ is the number of monomers per unit volume and b is the step length per monomer. We manipulate the expression for f_{int} to obtain:

$$f_{int} = \left(\sqrt{\frac{N\chi_{AB}}{6}}l_{01} + \sqrt{\frac{N\chi_{BC}}{6}}l_{03} + \sqrt{\frac{N\chi_{AC}}{6}}l_{05}\right) \times \frac{b\sqrt{N}}{\tilde{A}_T}. \tag{S7}$$

We next scale all lengths by $R = \sqrt{Nb^2}$, writing $L_{01} = l_{01}/R$ and writing $\tilde{A}_T = R^2 A_T$, as in the main paper, to obtain

$$f_{int} = \frac{1}{A_T}\left(\sqrt{\frac{N\chi_{AB}}{6}}L_{01} + \sqrt{\frac{N\chi_{BC}}{6}}L_{03} + \sqrt{\frac{N\chi_{AC}}{6}}L_{05}\right). \tag{S8}$$

We now define scaled surface tensions

$$\gamma_{IJ} = \sqrt{\frac{N\chi_{IJ}}{6}} \tag{S9}$$

to obtain the final expression for interfacial energy per chain in units of $k_B T$ for an SSP:

$$f_{int} = \frac{1}{A_T}\left(\gamma_{AB}L_{01} + \gamma_{BC}L_{03} + \gamma_{AC}L_{05}\right) \tag{S10}$$

The interfacial energy per chain is now in terms of the position coordinates of the nodes.

Stretching energy in an SSP

In this section, we work directly in scaled units, with lengths written in units of $R = \sqrt{Nb^2}$, and energies written in units of $k_B T$. The stretching free energy is determined for each triangle separately and then added up to find the total stretching energy per chain in an SSP. Node 0 is the core. The presence of a core introduces a logarithmic correction to the stretching

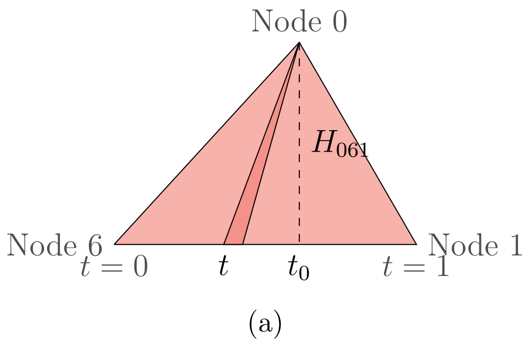

Figure S2: Example of two different possible geometries of triangle 061. The base length L_{061} is parametrized by t. The height H_{061} of the triangle is marked in both cases, and it intersects the base at t_0. The point of intersection can be within the triangle, and in (a), or outside the triangle, as in (b).

free energy. The calculation of the stretching free energy per chain is demonstrated by considering triangle 061, between Nodes 1, 6, and 0, of monomer type A.

The triangle 061 can have any triangular configuration. The perpendicular height of the triangle can intersect the base either inside or outside the triangle, as illustrated in fig. S2. The location of the wedge along the base of the triangle is parameterized by a variable t, which can take values from 0 to 1, between nodes 6 ($t = 0$) and 1 ($t = 1$). To determine the stretching free energy of the triangle, we calculate the stretching free energy for the wedge and sum over the length L_{061}.

In vector notation the nodes are written as $\vec{r}_0 = [x_0, y_0]^T$, $\vec{r}_1 = [x_1, y_1]^T$ and $\vec{r}_6 = [x_6, y_6]^T$. The perpendicular from node 0 intersects the base at $\vec{r}_h$, which is at a point parameterized by t_0, so $\vec{r}_h = \vec{r}_6 + t_0\,(\vec{r}_1 - \vec{r}_6)$. Since $(\vec{r}_h - \vec{r}_0)$ is perpendicular to $(\vec{r}_6 - \vec{r}_1)$, the dot product between these is zero, which gives t_0:

$$t_0 = \frac{(\vec{r}_6 - \vec{r}_0) \cdot (\vec{r}_6 - \vec{r}_1)}{|\vec{r}_6 - \vec{r}_1|^2}.$$

Now that t_0, and hence $\vec{r}_h$, is obtained in terms of the positions of nodes 0, 1 and 6, the height of the triangle is $H_{061} = |\vec{r}_h - \vec{r}_0|$ and the length of the base of the triangle is $L_{061} = |\vec{r}_6 - \vec{r}_1|$.

With t_0 as given above, the height of a wedge $H(t)$ at any t is then

$$H(t) = \sqrt{((t - t_0)L_{061})^2 + H_{061}^2}. \tag{S11}$$

We now consider the triangle in fig. S2(a) to be extended in the third dimension to a depth D (in scaled length units), giving a volume $A_{061}D$. The SSP, with area A_T, has corresponding volume $A_T D$. A single ABC star terpolymer occupies volume V_p (in scaled units), so the total number of ABC star terpolymers in the SSP is $A_T D/V_p$. An A-block occupies volume $\phi_A V_p$, so the total number of A-blocks in the triangle is $A_{061}D/(\phi_A V_p)$. The number of A-blocks in the wedge spanned by increment dt is $A_{061}D/(\phi_A V_p) \times dt$, since the area of a wedge of width dt is $A_{061}dt$, independent of the position of the wedge. The stretching energy per A-block in a wedge, as given in eq. (2), is

$$f_{chain}(H(t), \phi_A) = \frac{3}{4\phi_A} H(t)^2 \log(cH(t)^2) = \frac{3}{4\phi_A} F_{chain}(H(t)), \tag{S12}$$

where $c = R^2/R_{core}^2$ and $F_{chain}(H) = H^2 \log(cH^2)$. Multiplying by the number of A chains in a wedge and integrating over all wedges in the triangle gives the total free energy for triangle 061:

$$\frac{A_{061}D}{\phi_A V_p} \int_0^1 f_{chain}(H(t), \phi_A) dt.$$

The contribution from triangle 061 to the stretching energy per chain in the SSP can be obtained by dividing this by the total number of polymers in the SSP, giving:

$$f_{061} = \frac{V_p}{A_T D} \frac{A_{061}D}{\phi_A V_p} \int_0^1 f_{chain}(H(t), \phi_A) dt = \frac{A_{061}}{\phi_A A_T} \int_0^1 f_{chain}(H(t), \phi_A) dt. \tag{S13}$$

We substitute from eq. (S12) using the wedge height in eq. (S11) to give

$$f_{061} = \frac{3A_{061}}{4\phi_A^2 A_T} \int_0^1 F_{chain} \left(\sqrt{((t - t_0)L_{061})^2 + H_{061}^2} \right) dt, \tag{S14}$$

where $F_{chain}(H) = H^2 \log(cH^2)$.

The integral above is of the general form,

$$I_{061}(X, Y, t_0) = \int_0^1 (X(t - t_0)^2 + Y) \log(c(X(t - t_0)^2 + Y))dt, \tag{S15}$$

where $X = L_{061}^2$, $Y = H_{061}^2$ and $H(t) = X(t - t_0)^2 + Y$. This integral can be evaluated explicitly:

$$\begin{aligned} I_{061} =& \log(c(X(1 - t_0)^2 + Y)) \left(\frac{X(1 - t_0)^3}{3} + Y(1 - t_0) \right) + \\ & \log(c(Xt_0^2 + Y)) \left(\frac{Xt_0^3}{3} + Yt_0 \right) - \frac{2X}{9} \left((1 - t_0)^3 + t_0^3 \right) - \frac{4Y}{3} + \\ & \frac{4Y}{3} \sqrt{\frac{Y}{X}} \left(\arctan \sqrt{\frac{X}{Y}}(1 - t_0) + \arctan \sqrt{\frac{X}{Y}} t_0 \right). \end{aligned} \tag{S16}$$

Hence, for triangle 061, the stretching free energy per chain in units of k_BT is

$$f_{061} = \frac{3}{4} \frac{A_{061}}{\phi_A^2 A_T} I_{061}(L_{061}^2, H_{061}^2, t_0), \tag{S17}$$

where t_0 is given above in terms of the positions of nodes 0, 6 and 1.

The total stretching free energy per chain f_{str} of an SSP is given by summing the equivalent expressions for all six triangles, recalling that triangles 061 and 056 have monomer A, triangles 023 and 012 have monomer B, triangles 045 and 034 have monomer C, and that t_0 will be different for each triangle. The outcome is:

$$f_{str} = f_{061} + f_{056} + f_{045} + f_{034} + f_{023} + f_{012}. \tag{S18}$$

Core contributions to free energy

Since we expect the core region to be of order monomer dimensions, the exact details must depend on the specific chemistry, both of the monomer type in each of the three arms,

and of the chemical unit used to form the branch point itself. It is not possible to develop a "universal" theory. However, we can make reasonable assumptions to arrive at a plausible first order description of the core. We assume that the chains in the core region are sufficiently closely packed together so that the arms are forced to exit the core region as quickly as possible, i.e., they are strongly stretched away from the core at the monomer scale. Thus, if the core region contains $N_{core} \ll N$ monomers per chain, we expect $R_{core} \approx N_{core} b/3$ for a three arm star.

Now, consider an SSP of area $\tilde{A}_T = R^2 A_T$ (scaled by length $R = \sqrt{Nb^2}$) and depth d in the third dimension, and so of volume $\tilde{A}_T d$. The volume per chain is Nv_0, where v_0 is the volume of a single monomer unit. Then the number of chains in the SSP is,

$$\frac{\tilde{A}_T d}{N v_0}.$$

But, the core region contains N_{core} monomers per chain, each of volume v_0, so the core volume must be:

$$\frac{\tilde{A}_T d}{N v_0} N_{core} v_0 = \frac{\tilde{A}_T d N_{core}}{N}.$$

But the core volume is also $\pi R_{core}^2 d$, so we find:

$$\pi R_{core}^2 d = \frac{\tilde{A}_T d N_{core}}{N},$$
$$R_{core}^2 = \frac{\tilde{A}_T N_{core}}{\pi N}.$$

But we also have $R_{core} \approx N_{core} b/3$, and so:

$$\frac{N_{core}^2 b^2}{9} = \frac{\tilde{A}_T N_{core}}{\pi N},$$
$$N_{core} = \frac{9\tilde{A}_T}{\pi N b^2} = \frac{9 A_T}{\pi}$$

and hence:

$$R_{core} = \frac{3A_T b}{\pi}.$$

As A_T increases, the number of chains per unit length of the core also increases, so that the radius of the core (in which chains are stretched to monomer level by chain packing) must increase. Hence, we find:

$$c = \frac{R^2}{R_{core}^2} \approx \frac{\pi^2 N}{9A_T^2}, \tag{S19}$$

i.e., the value of c used in the equations for the stretching free energy is not constant but varies with the SSP area, and depends on the degree of polymerization N.

The chains inside the core region are strongly stretched to the monomer level, and we estimate the energy for this to be of order $k_B T$ per monomer, giving an energy per chain (in units of $k_B T$) of:

$$f_{st,core} = s_{core} N_{core} = s_{core} \frac{9A_T}{\pi},$$

where s_{core} is a parameter expected to be order one.

Monomers within the core are also necessarily brought into close proximity, and we expect the number of monomers of each type (A, B or C) in the core to be approximately equal. Hecne, we assume the composition of the core is approximately $\phi_A = \phi_B = \phi_C = \frac{1}{3}$, giving an energy per chain of:

$$\begin{aligned} f_{int,core} &= N_{core}(\chi_{AB}\phi_A\phi_B + \chi_{BC}\phi_B\phi_C + \chi_{AC}\phi_A\phi_C) \\ &= \frac{9A_T}{\pi}\left(\frac{\chi_{AB} + \chi_{BC} + \chi_{AC}}{9}\right). \end{aligned}$$

Adding these together gives our proposed core energy, of:

$$f_{core} = f_{st,core} + f_{int,core} = \frac{9A_T}{\pi}\left(s_{core} + \frac{\chi_{AB} + \chi_{BC} + \chi_{AC}}{9}\right). \tag{S20}$$

We add the stretching free energy eq. (S18) to the interfacial free energy eq. (S10) and

core energy eq. (S20) to obtain the total free energy per chain of an SSP f_c, in units of $k_B T$,

$$f_c = f_{int} + f_{str} + f_{core}. \tag{S21}$$

This total free energy is now given explicitly in terms of the positions of the six nodes, the monomer compositions (ϕ_A, ϕ_B, ϕ_C) (with $\phi_A + \phi_B + \phi_C = 1$), the Flory interaction parameters and the degree of polymerization.

Phase diagrams with different values of N

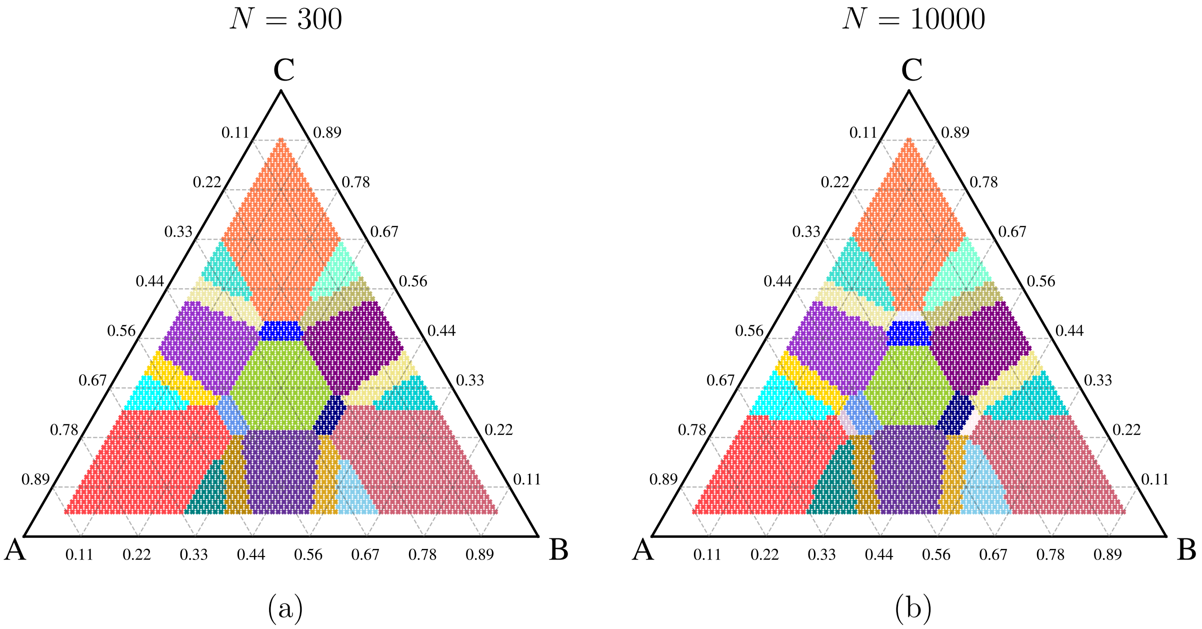

Figure S3: Phase diagrams with different values of N: (a) $N = 300$; (b) $N = 10000$. Compare with diagram for $N = 1000$, shown in fig. 6(b). The data for this figure is available from.[1]

Figure S3 shows phase diagrams with $N = 300$ and $N = 10000$. Compared to $N = 1000$ (fig. 6b), the overall placement of regions with stable morphologies is qualitatively unchanged, though there are subtle changes in the areas covered by each morphology. The $[10.6.4; 10.4.6; 10.6.6]$ morphology, masked by L+C in fig. 12(b), appears in phase diagram corresponding to higher degree of polymerization $N = 10,000$ in fig. S3.(b), with two rows of $[10.6.4; 10.4.6; 10.6.6]$ (light blue regions next to orange lamellar L+C regions).

References

(1) Joseph, M.; Read, D. J.; Rucklidge, A. M. Dataset for "A flexible implementation of strong segregation theory for two dimensional ABC star terpolymer morphologies". University of Leeds Data Repository `https://doi.org/10.5518/1879`, 2026.
